# Supplementary material for: Opposing Regulation of Cocaine Seeking by Glutamate and GABA Neurons in the Ventral Pallidum
Source: Cell Rep. Author manuscript; Available in PMC 2020 Feb 27. (PMC7045305; doi:10.1016/j.celrep.2020.01.023)
Supplement: 1 [file NIHMS1560164-supplement-1.pdf]

Cell Reports, Volume *30*

Supplemental Information

Opposing Regulation of Cocaine Seeking by Glutamate and GABA Neurons in the Ventral Pallidum

Jasper A. Heinsbroek, Ana-Clara Bobadilla, Eric Dereschewitz, Ahlem Assali, Reda M. Chalhoub, Christopher W. Cowan, and Peter W. Kalivas

SUPPLEMENTAL INFORMATION

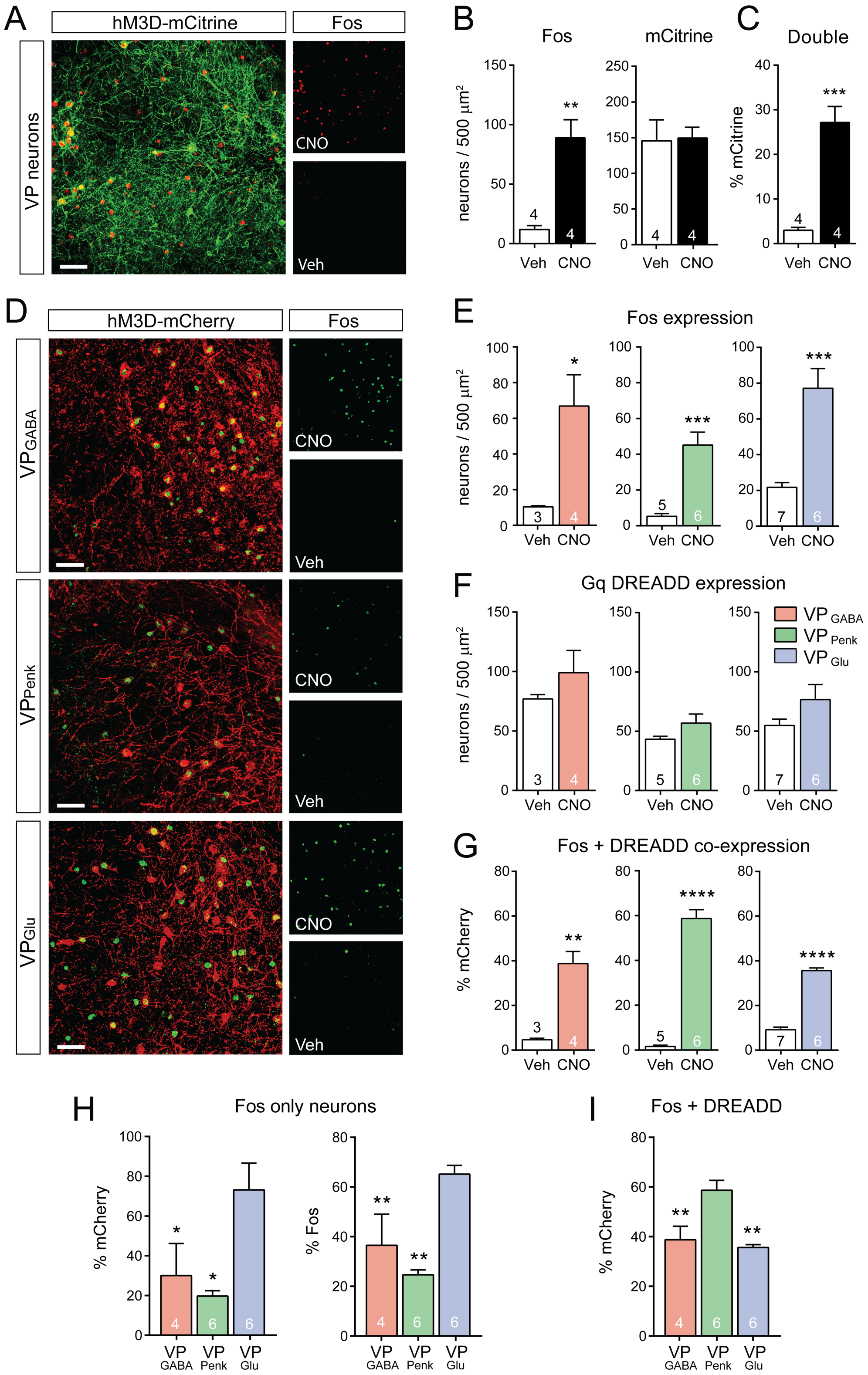

Figure S1. Gq DREADD Activation by CNO Induces Fos in VP Neurons, Related to Figure 2 and 3. (A) Representative micrograph showing that Fos expression induced by CNO co-localizes with Gq DREADD (mCitrine) expression in the VP. Inserts to the right show Fos expression following an injection of CNO (top) or vehicle (Veh, bottom). Bar= 50 um. (B) CNO significantly induces Fos in the VP of animals infected with Gq DREADD. (C) CNO induces Fos in Gq DREADD virus (mCitrine) expressing neurons. (D) Representative images showing Fos and Gq DREADD co-expression in VP_{GABA}, VP_{Penk} and VP_{Glu}. Inserts on the right show Fos induction in an animal injected with CNO (top) or Veh (bottom). Bar= 50 um. (E) Gq DREADD activation significantly induced Fos in all VP neuronal subtypes. (F) Gq DREADD (mCherry) expression levels were similar between CNO and Veh treated mice. (G) CNO significantly induced Fos in virus infected neurons across all cell-type. (H) Non-virus infected neurons were Fos activated in significantly higher numbers following stimulation of VP_{Glu} compared to VP_{GABA} or VP_{Penk} neurons (counts normalized to total virus expression or total Fos expression). (I) CNO induces Fos in a larger fraction of VP_{Penk} compared to VP_{GABA} or VP_{Glu} neurons. n in bars = number of animals. Data represented as mean ± SEM. * $p<0.05$, ** $p<0.01$, *** $p<0.001$, **** $p<0.0001$ (for H comparing VP_{GABA} and VP_{Penk} to VP_{Glu}, for I comparing VP_{GABA} and VP_{Glu} to VP_{Penk}).

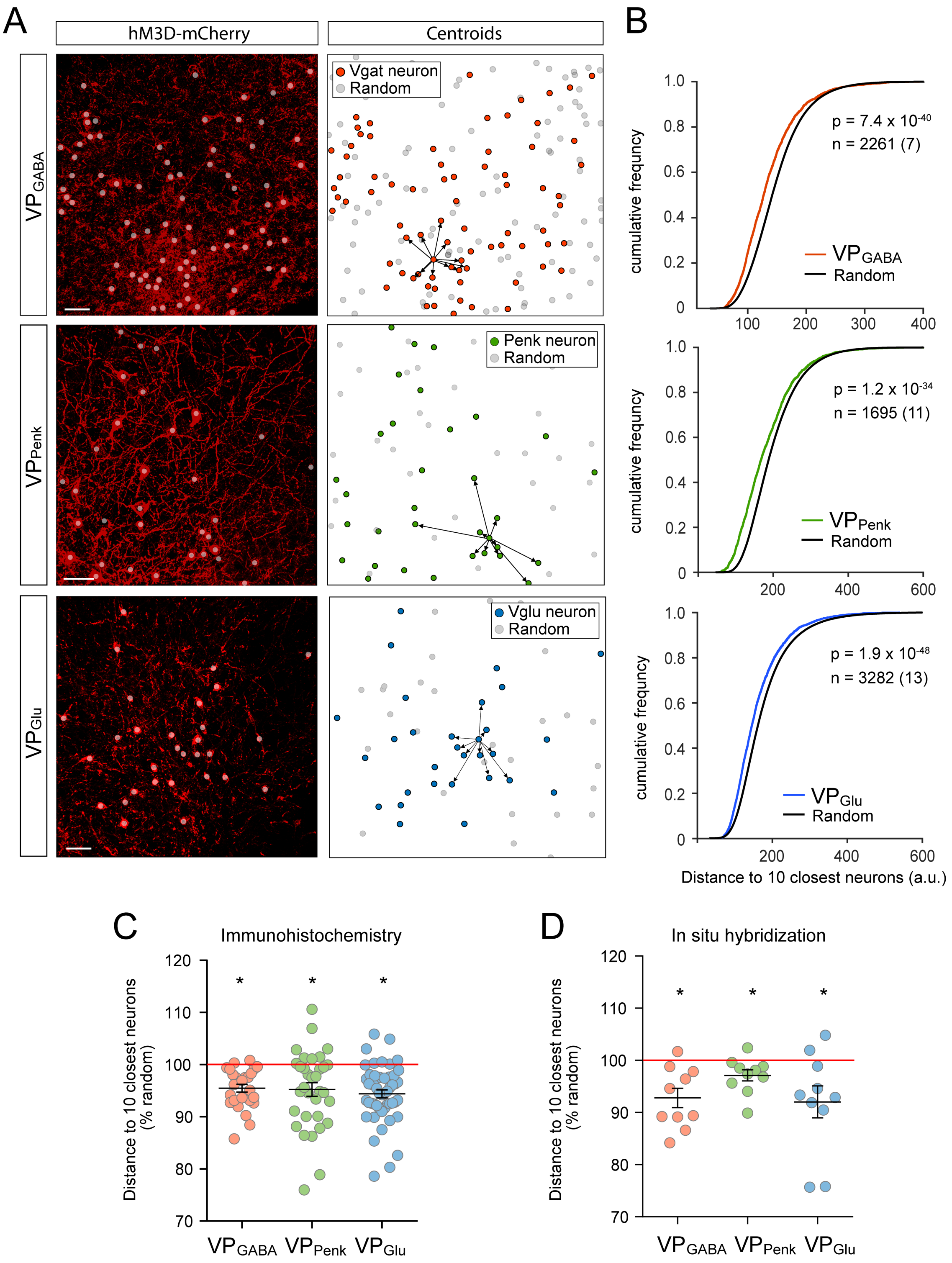
A
hM3D-mCherry
Centroids
VPGABA
Vgat neuron
Random
VPPenk
Penk neuron
Random
VPGlu
Vglu neuron
Random
B
cumulative frequncy
p = 7.4 x 10-40
n = 2261 (7)
VPGABA
Random
p = 1.2 x 10-34
n = 1695 (11)
VPPenk
Random
p = 1.9 x 10-48
n = 3282 (13)
VPGlu
Random
Distance to 10 closest neurons (a.u.)
C
Immunohistochemistry
Distance to 10 closest neurons (% random)
VPGABA VPPenk VPGlu
D
In situ hybridization
Distance to 10 closest neurons (% random)
VPGABA VPPenk VPGlu

Figure S2. VP Subpopulations are Organized in Clusters, Related to Figure 3. (A) Left: micrographs showing mCherry expression in subpopulations of VP neurons with overlaid centroids for each cell (white dots). Right: Example centroid maps (color) for representative image stacks of each cell type combined with a randomly generated centroid map (grey) containing the same number of datapoints, as well as an example showing the 10 nearest neighbors of a single cell. (B) Cumulative frequency distribution for the distances of each VP cell to its 10 nearest neighbors, and the equivalent distance between random datapoints. VP subpopulations were significantly more clustered than random data (2-sample Kolgomorov-Smirnov test) n= total cell# over (mouse #) collected in 3-4 image stacks per animal. (C) K-nearest neighbor distance for all cells per image stack (normalized to the equivalent distance between random datapoints in that stack). VP neurons identified by mCherry expression (D) or RNA expression (E) were significantly more organized as clusters than random data (one-sample t-test). Error bars represent SEM. * $p < 0.05$

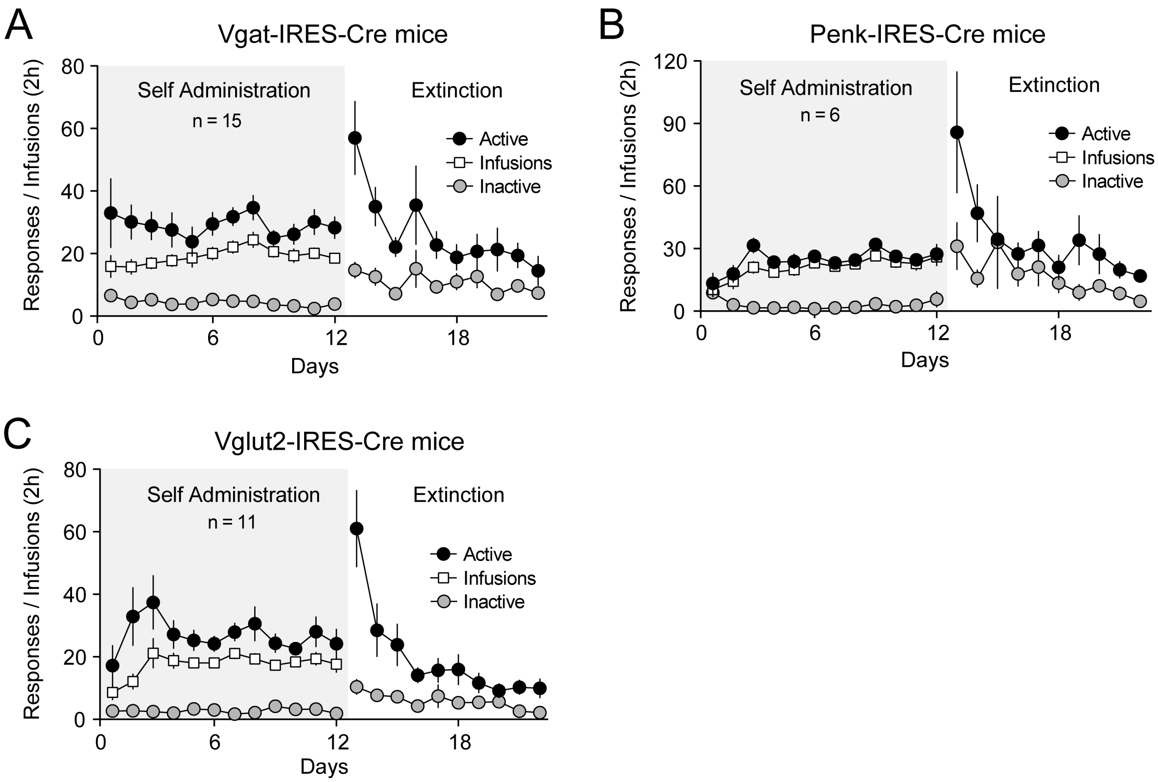

Figure S3. Cocaine Self-administration and Extinction for Gq DREADD Experiment, Related to Figure 3. (A) Vgat-IRES-Cre mice, (B) Penk-IRES-Cre mice, and (C) Vglut2-IRES-Cre lines acquired stable self-administration after 3d and extinguished operant responding in the absence of cocaine. Data represented as mean ± SEM.

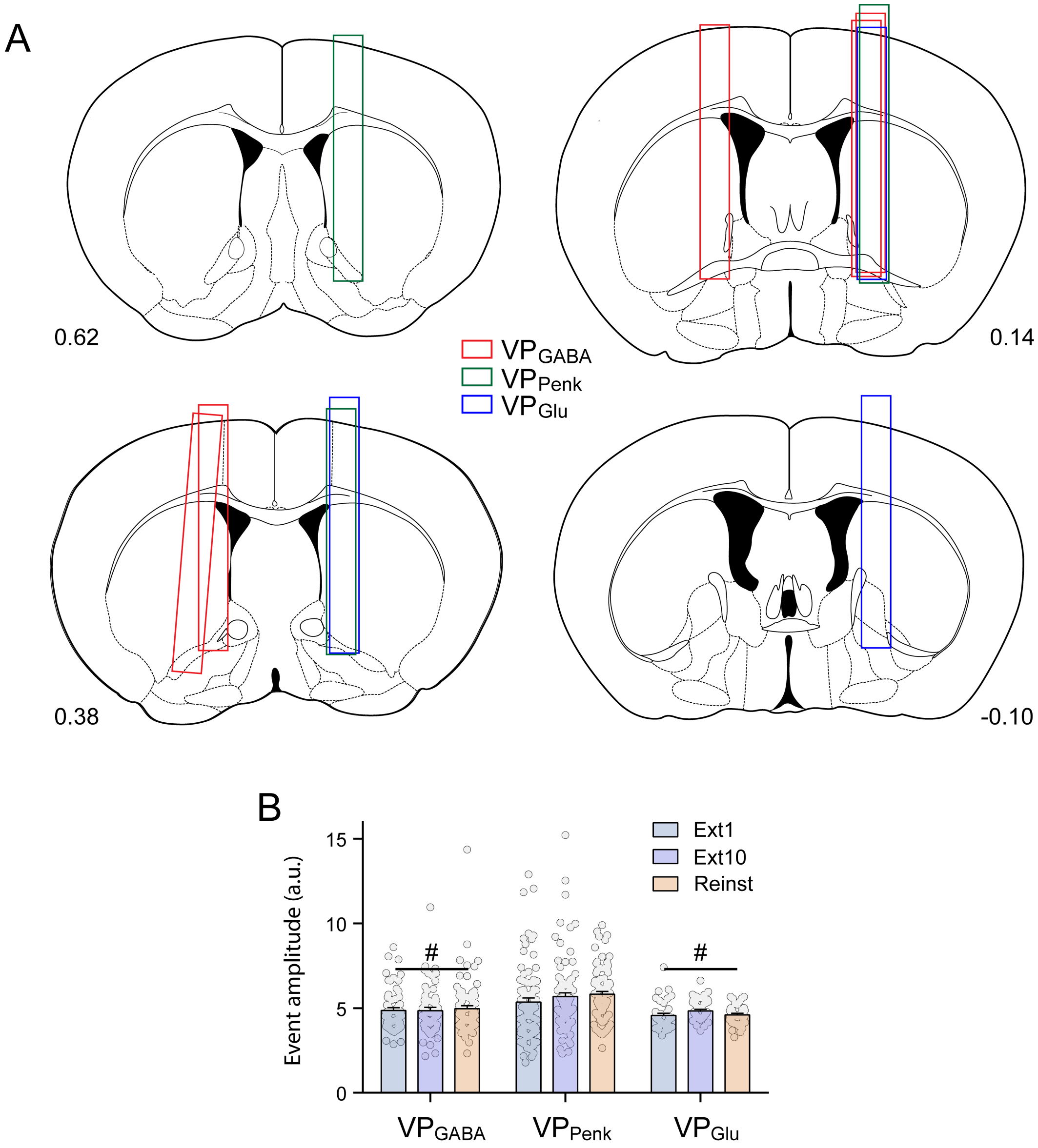

Figure S4. GRIN Lens Placements and Ca^{2+} Event Amplitudes, Related to Figure 4 and 5. (A) Placements of GRIN lenses for recordings of the different VP subpopulations of neurons. Numbers represent anteroposterior coordinates relative to Bregma. (C) Ca^{2+} event amplitude does not differ across conditions, but the Ca^{2+} event amplitude of VP_{Penk} neurons is significantly higher than that of VP_{Glu} and VP_{GABA} neurons. Data represented as mean ± SEM. # $p < 0.05$ compared to VP_{Penk}.

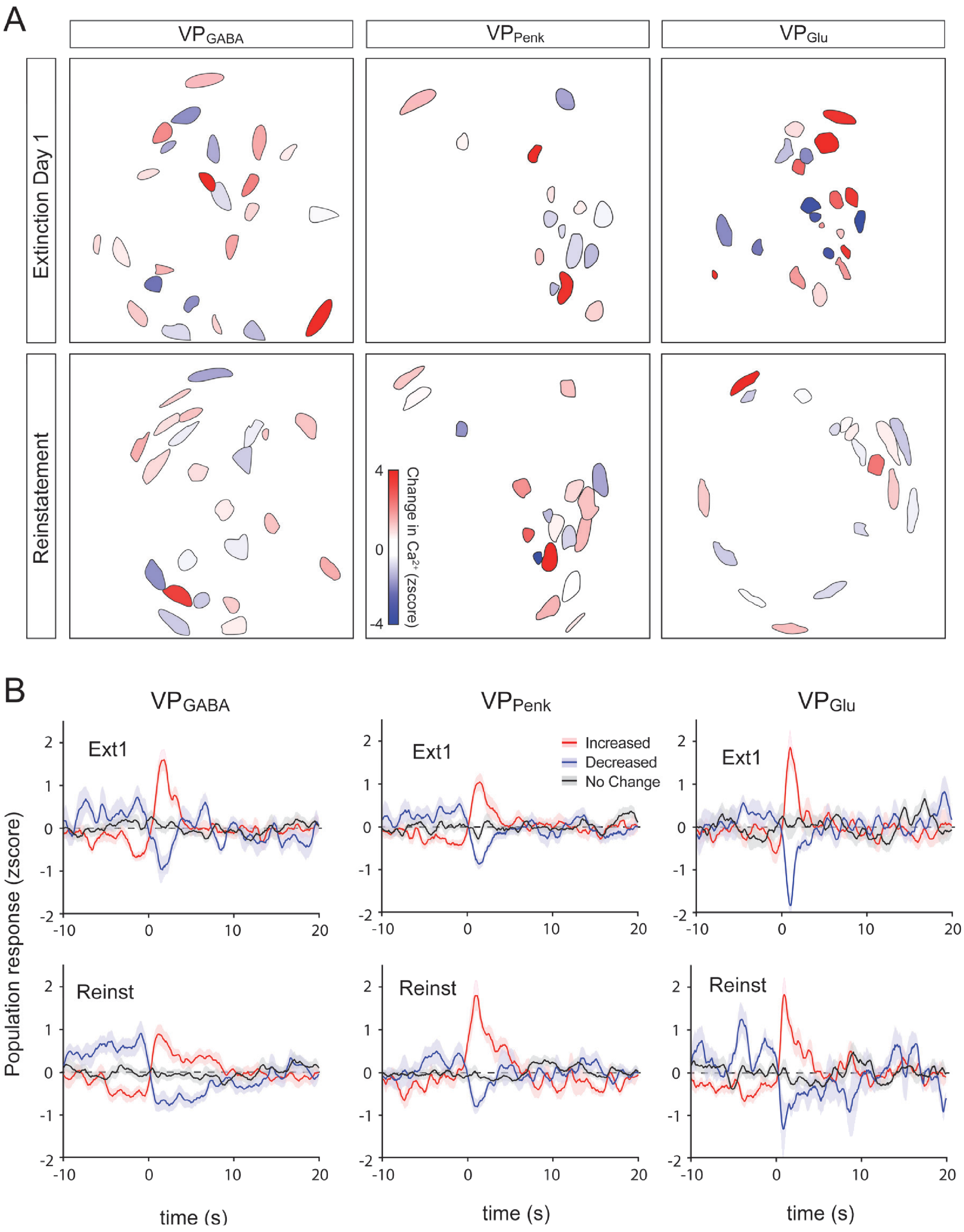
A
VP_{GABA}
VP_{Penk}
VP_{Glu}
Extinction Day 1
Reinstatement
Change in Ca^{2+} (zscore)
4
0
-4
B
Population response (zscore)
Ext1
Reinst
Increased
Decreased
No Change
time (s)
2
1
0
-1
-2
-10
0
10
20

Figure S5. Populations of VP Neurons Subdivided by Increased or Decreased Activity Patterns, Related to Figure 5. (A) Example cell maps of the distinct VP neurons isolated from Ca^{2+} videos, pseudocolored by activity profile showing neurons responding with increased or decreased activity during the first 2s following a nosepoke are spread heterogeneously throughout the VP. (B) Population responses for all cell types and conditions separated by neurons that show increased, decreased or unchanged Ca^{2+} activity 2s following a nosepoke. Data represented as mean (lines) ± SEM (shading).
