# Supplementary material for: Opposing Regulation of Cocaine Seeking by Glutamate and GABA Neurons in the Ventral Pallidum
Source: Cell Rep. Author manuscript; Available in PMC 2020 Feb 27. (PMC7045305; doi:10.1016/j.celrep.2020.01.023)
Supplement: 2 [file NIHMS1560164-supplement-2.pdf]

Cell Reports

Article

Opposing Regulation of Cocaine Seeking by Glutamate and GABA Neurons in the Ventral Pallidum

Graphical Abstract

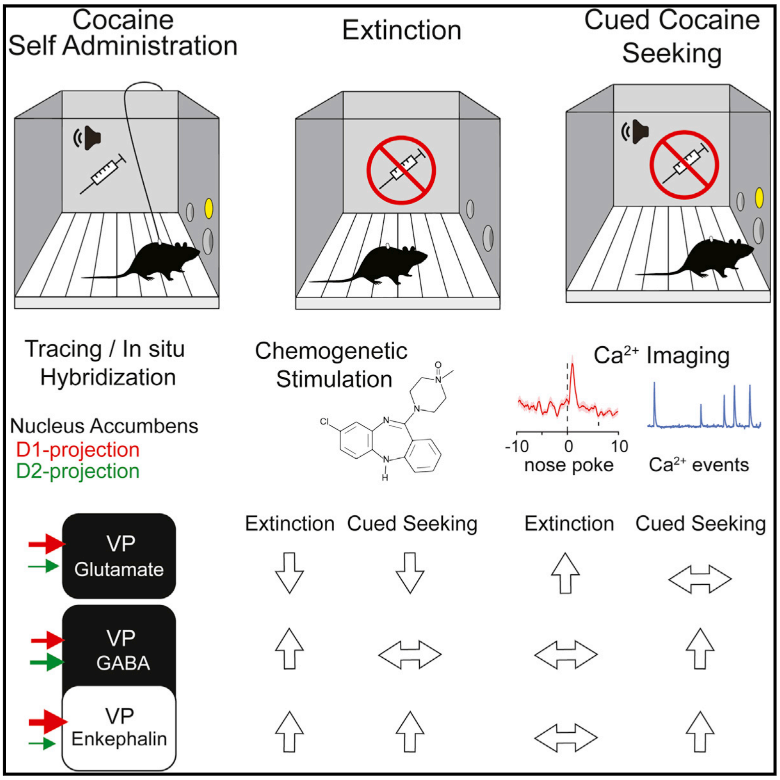

Authors

Jasper A. Heinsbroek, Ana-Clara Bobadilla, Eric Dereschewitz, Ahlem Assali, Reda M. Chalhoub, Christopher W. Cowan, Peter W. Kalivas

Correspondence

jasper.heinsbroek@ucdenver.edu (J.A.H.),
kalivasp@musc.edu (P.W.K.)

In Brief

Heinsbroek et al. show that glutamate and GABA neurons in ventral pallidum differentially regulate cued cocaine seeking. Calcium activity in glutamate neurons increases when mice refrain from cocaine seeking. Activating glutamate neurons inhibits cocaine seeking. Calcium activity increases in GABA neurons during cocaine seeking, and activating GABA or enkephalin neurons induces cocaine seeking.

Highlights

- Cocaine seeking is potentiated by stimulating GABAergic ventral pallidum neurons
- Cocaine seeking is reduced by stimulating glutamatergic ventral pallidal neurons
- Glutamate and enkephalin neurons are differentially innervated by accumbens neurons
- Calcium is increased during seeking in GABA and during extinction in glutamate neurons

Heinsbroek et al., 2020, Cell Reports *30*, 2018–2027
February 11, 2020 © 2020 The Author(s).
https://doi.org/10.1016/j.celrep.2020.01.023

OPEN ACCESS
CellPress

Cell Reports
Article

Opposing Regulation of Cocaine Seeking by Glutamate and GABA Neurons in the Ventral Pallidum

Jasper A. Heinsbroek,[1,*] Ana-Clara Bobadilla,[2] Eric Dereschewitz,[2] Ahlem Assali,[2] Reda M. Chalhoub,[2] Christopher W. Cowan,[2] and Peter W. Kalivas[2,3,*]
[1]Department of Anesthesiology, University of Colorado Denver, Anschutz Medical Center, Aurora, CO 80045, USA
[2]Department of Neuroscience, Medical University of South Carolina, Charleston, SC 29425, USA
[3]Lead Contact
*Correspondence: jasper.heinsbroek@ucdenver.edu (J.A.H.), kalivasp@musc.edu (P.W.K.)
https://doi.org/10.1016/j.celrep.2020.01.023

SUMMARY

Projections from the nucleus accumbens to the ventral pallidum (VP) regulate relapse in animal models of addiction. The VP contains GABAergic (VP_{GABA}) and glutamatergic (VP_{Glu}) neurons, and a subpopulation of GABAergic neurons co-express enkephalin (VP_{Penk}). Rabies tracing reveals that VP_{Glu} and VP_{Penk} neurons receive preferential innervation from upstream D1- relative to D2-expressing accumbens neurons. Chemogenetic stimulation of VP_{Glu} neurons inhibits, whereas stimulation of VP_{GABA} and VP_{Penk} neurons potentiates cocaine seeking in mice withdrawn from intravenous cocaine self-administration. Calcium imaging reveals cell type-specific activity patterns when animals learn to suppress drug seeking during extinction training versus engaging in cue-induced cocaine seeking. During cued seeking, VP_{GABA} neurons increase their overall activity, and VP_{Penk} neurons are selectively activated around nose pokes for cocaine. In contrast, VP_{Glu} neurons increase their spike rate following extinction training. These data show that VP subpopulations differentially encode and regulate cocaine seeking, with VP_{Penk} and VP_{GABA} neurons facilitating and VP_{Glu} neurons inhibiting cocaine seeking.

INTRODUCTION

The interconnected nuclei of the ventral basal ganglia regulate motivated behavior and reward learning. Within this network, activity in ventral pallidum (VP) neurons is necessary for motivated drug seeking in animal models of addiction for all known drugs of abuse (Farrell et al., 2019; Heinsbroek et al., 2017; Mahler et al., 2014; McFarland and Kalivas, 2001; Rogers et al., 2008; Root et al., 2015). Recent studies challenge a traditional view that the VP is a GABAergic relay structure, by demonstrating complex cell type-specific information processing in pallidal brain regions (i.e., VP and globus pallidus) (Beier et al., 2017; Knowland et al., 2017; Ottenheimer et al., 2018; Richard et al., 2016; Wallace et al., 2017). Specifically, the VP contains two distinct projection neuron subtypes that constitute >95% of all VP neurons, including glutamatergic (VP_{Glu}) and GABAergic (VP_{GABA}) neurons (Geisler et al., 2007; Hur and Zaborszky, 2005), and very few VP_{Glu} neurons co-express glutamate and GABA (Root et al., 2018; Tooley et al., 2018). Activation of VP_{Glu} neurons drives aversion (Faget et al., 2018; Tooley et al., 2018), whereas stimulating VP_{GABA} neurons motivates reward seeking (Faget et al., 2018; Zhu et al., 2017). These effects are mediated in part by projections to the ventral tegmental area (VTA) and lateral habenula (lHb) (Faget et al., 2018).

Recent studies suggest that VP_{GABA} neurons may be composed of multiple neuronal subtypes. For example, some globus pallidus GABAergic neurons expressing NK2 homeobox 1 project to canonical output structures, while other GABAergic neurons express enkephalin and forkhead box P2 and preferentially innervate the striatum (Dodson et al., 2015; Mallet et al., 2012). These pallidal GABAergic subpopulations have distinct electrophysiological properties and in dorsal striatum mediate different aspects of movement execution (Dodson et al., 2015; Mallet et al., 2016). Although globus pallidus is strongly linked to motor behavior, the VP is tied more closely to the motivation that guides motor behavior. Studies from our lab indicate that a subset of VP neurons express the neuropeptide enkephalin (Kalivas et al., 1993) and that VP enkephalin signaling is necessary for the reinstatement of cocaine seeking in an animal model of addiction (Tang et al., 2005). However, a role for any of these three VP subtypes (VP_{Glu}, VP_{GABA}, and VP enkephalin [VP_{Penk}] neurons) in cue-induced drug seeking (which models the ability of drug-associated stimuli to drive drug craving and relapse) remains unexplored.

The main input to VP neurons arises from the nucleus accumbens, and this pathway is composed of dopamine D1 and D2 receptor-expressing GABAergic medium spiny neurons (D1- and D2-MSNs) (Creed et al., 2016; Heimer, 1975; Kupchik et al., 2015; Matsui and Alvarez, 2018). D1 and D2 projections convey opposing information to the VP regarding drug seeking, with D1 input promoting cocaine behavioral sensitization and cocaine seeking and D2 input promoting cocaine withdrawal-induced anhedonia and extinguished cocaine seeking (Creed et al., 2016; Heinsbroek et al., 2017; Pardo-Garcia et al., 2019; Roberts-Wolfe et al., 2018). Withdrawal from cocaine with or without extinction training produces persistent enkephalinergic tone onto presynaptic μ opioid receptors located on D2-MSN

 © 2020 The Author(s).
This is an open access article under the CC BY license (http://creativecommons.org/licenses/by/4.0/).

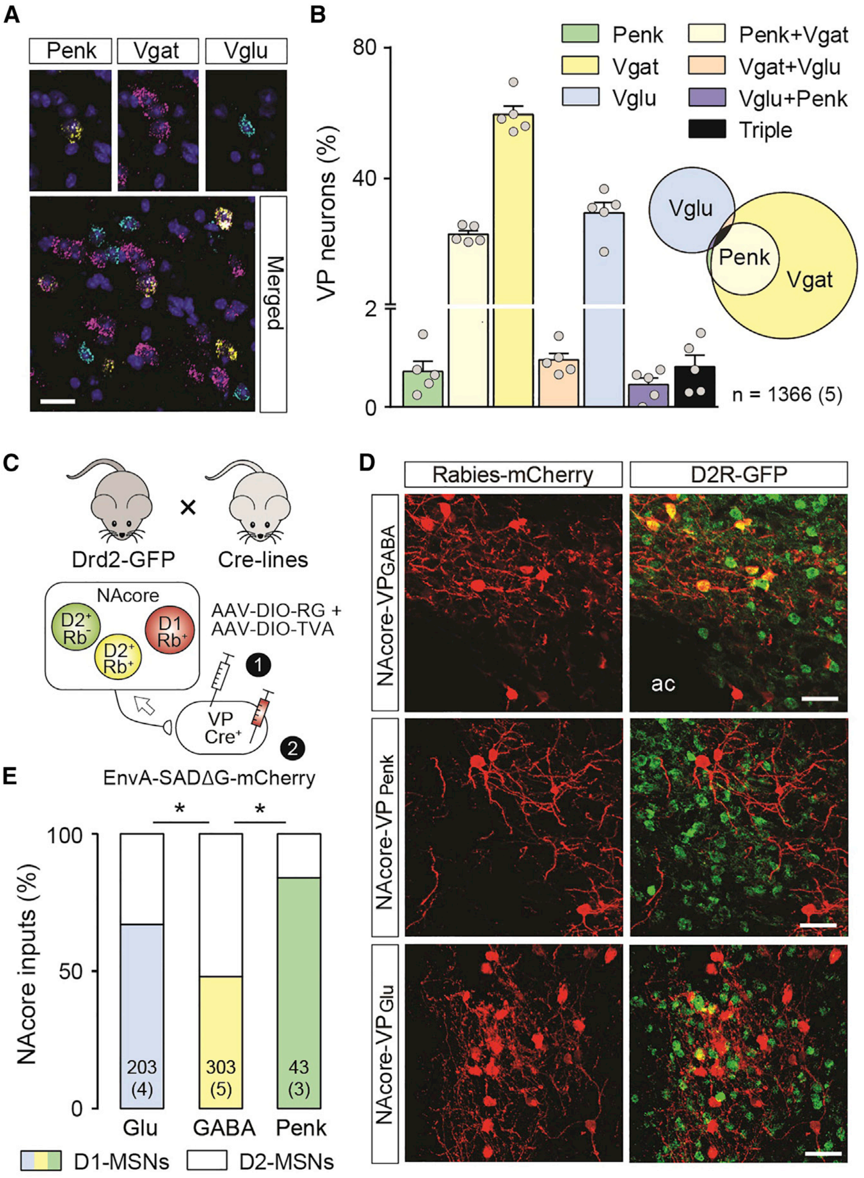

Figure 1. Relative Density of VP Neuronal Subtypes and Proportion of Inputs Derived from NAcore D1- and D2-MSNs

(A) Representative micrograph from the VP showing triple *in situ* hybridization of mRNA encoding Vglut2 (Slc17a6; turquoise), Penk (yellow), Vgat (slc32a1; pink), and a DAPI nuclear counterstain. Scale bar, 25 μm.

(B) Relative density of the combinations of Vglut2, Penk, and Vgat expression in VP neurons, shown both as a percentage of each cell type in a bar graph and in a proportional Venn diagram. n = cell number over (mouse number).

(C) Reporter mice used to characterize neurons upstream of the distinct VP populations as D1-MSNs (D2-GFP$^-$) or D2-MSN (D2-GFP$^+$) were generated by crossing Drd2-GFP mice with Vglut2, Vgat, and Penk Cre mouse lines (top). Helper viruses introduced rabies G-protein and the avian sarcomavirus leukosis receptor A (TVA) into distinct VP starter cells, followed by pseudotyped replication-deficient rabies.

(D) Representative micrographs showing rabies-infected D1-MSN (red) and D2-MSN (yellow) in the NAcore. Scale bar, 25 μm.

(E) VP_{Glu} and VP_{Penk} neurons are preferentially innervated by D1-MSN. n in bars = cell number over (mouse number).

Data in bars are presented as mean ± SEM. Chi-square tests with Bonferroni-adjusted p values for repeated testing. *p < 0.05.

terminals in the VP, and the elevated μ opioid tone reduces D2-MSN activity, thereby promoting cocaine seeking (Heinsbroek et al., 2017; Kupchik et al., 2014). However, the relative innervation by D2- versus D1-MSNs onto the different VP neuronal subpopulations is unknown.

Here we describe the results form a series of experiments conducted to dissect the subcircuit connectivity and functional roles of the VP_{Penk}, VP_{Glu}, and VP_{GABA} subtypes on cocaine seeking after extinction training in self-administering mice.

RESULTS

VP Neuronal Subtypes: Anatomical Distinctions

We used *in situ* hybridization (RNAscope) to quantify neuron subtype density in subcommissural dorsal VP (dVP)—VP_{Glu} (23%), VP_{GABA} (73%), and VP_{Penk} (16%)—and found that >90% of VP_{Penk} and <5% of VP_{Glu} neurons co-expressed the vesicular GABA transporter (Vgat) (Figures 1A and 1B). The dVP is innervated by the core subcompartment of the accumbens (NAcore) (Heimer et al., 1991), and we used a retrograde rabies labeling strategy to determine the relative innervation of the distinct dVP cell types by NAcore D1- and D2-MSNs. D2-eGFP reporter mice were crossed with mice expressing Cre recombinase selectively in each VP cell type (Vglut2-IRES-Cre, Vgat-IRES-Cre, or Penk-IRES-Cre), and VP cells were transduced with rabies helper virus constructs (avian sarcoma/leukosis virus receptor A and rabies glycoprotein) followed by a pseudotyped replication-deficient rabies-mCherry vector (Figure 1C). Rabies tracing showed that although VP_{GABA} neurons were innervated equally by D1- and D2-MSNs, both VP_{Glu} and VP_{Penk} neurons had more D1-MSN-labeled afferents (Figures 1D and 1E; VP_{Glu} versus VP_{GABA} chi-square = 12.3, p = 2.3 × 10^{-8}; VP_{Penk} versus VP_{GABA} chi-square = 10.9, p = 6.3 × 10^{-4}; with Bonferroni adjustment for multiple comparisons). Although we did not distinguish between enkephalin-expressing and non-enkephalin-expressing VP_{GABA} neurons, given that 22% of VP_{GABA} neurons are VP_{Penk} neurons (Figure 1B), and that 84% of NAcore inputs onto those neurons arise from D1-MSNs (Figure 1E), the remaining 78% of

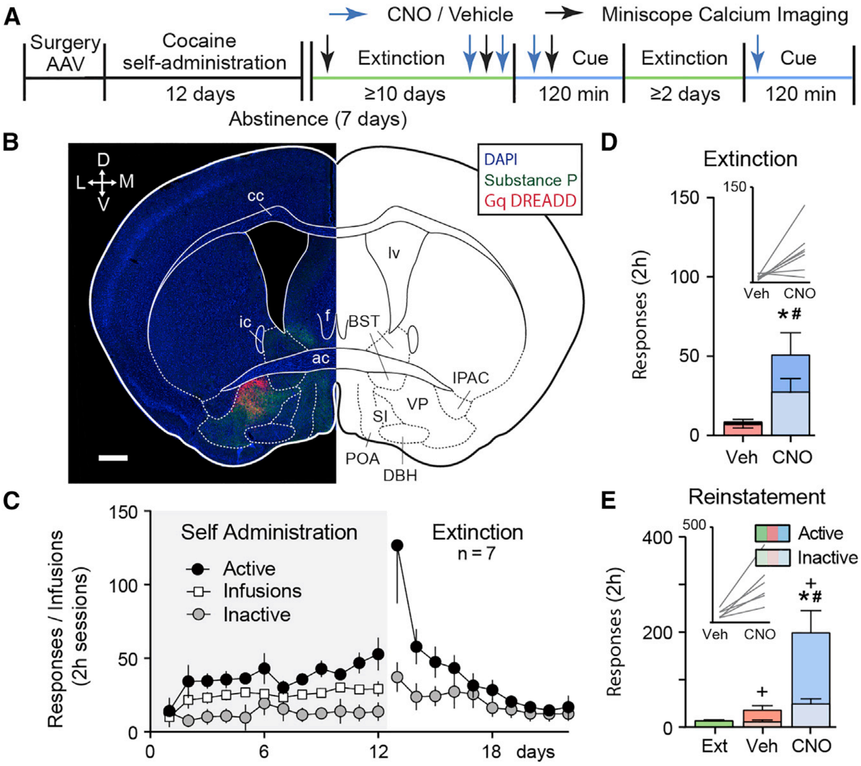

Figure 2. Chemogenetic Stimulation of VP Neurons Augments Extinction- and Cue-Induced Cocaine Seeking

(A) Outline of the protocol used in the chemogenetic and miniscope Ca^{2+} imaging experiments.

(B) Representative example of non-selective Gq-DREADD expression in the VP. ac, anterior commissure; BST, bed nucleus of the stria terminalis; cc, corpus callosum; DBH, diagonal horizontal band of Broca; f, fornix; ic, interior commissure; IPAC, interstitial nucleus of the posterior anterior commissure; lv, lateral ventricle; POA, preoptic area; SI, substantia innominata. Scale bar, 500 um. mCitrine expression is pseudocolored red for clarity. Substance P (green) was used as a counterstain to outline the borders of the VP.

(C) Cocaine self-administration and extinction in wild-type mice expressing Gq-DREADD in VP neurons.

(D and E) Simultaneously stimulating all VP neurons prior to a late extinction session (Ext) (D) or cue seeking in mice transduced with Gq DREADD in VP neurons (E) increased cocaine seeking.

Data are presented as mean ± SEM. *$p < 0.05$ comparing vehicle and CNO (active), +$p < 0.05$ comparing extinction and cue seeking (active), and #$p < 0.05$ comparing vehicle and CNO (inactive).

enkephalin-negative VP_{GABA} would be preferentially (62%) innervated by D2-MSNs.

VP Neuronal Subtypes: Distinct Roles in Extinction and Cued Cocaine Seeking

Activation of D1-MSNs in NAcore and their projections to the dVP are necessary for cocaine-associated cues to initiate drug seeking in rodents after extinction training (Pardo-Garcia et al., 2019; Stefanik et al., 2013). Given the predominance of D1-MSN inputs to VP_{Glu} and VP_{Penk} neurons (see Figure 1), we used a chemogenetic strategy to activate the different VP neuronal subtypes in Cre mice (Vglut2-IRES-Cre, Penk-IRES-Cre, and Vgat-IRES-Cre). Mice were allowed to self-administer cocaine for 12 days with a light/tone cue pairing to each cocaine infusion and then underwent $\geq$10 days of extinction training without cue presentation (Figures 2A–2C and S3). At the time of jugular catheter surgery, an adeno-associated virus (AAV) harboring a floxed or non-floxed Gq-coupled designer receptor activated exclusively by designer drugs (Gq-DREADD; AAV2-hSyn-DIO-hM3D-mCherry or AAV2-hSyn-hM3D-mCitrine) was microinjected into the VP (Figures 2B, and 3A, S1, and S2). Gq-DREADD was activated using a systemic injection of clozapine-N-oxide (CNO; 1 mg/kg, intraperitoneal [i.p.]) prior to a late extinction session or before a cue-induced cocaine seeking test (see Figure 2A for experimental timeline). Vehicle (Veh) and CNO were given in a randomized counter-balanced design, separated by $\geq$2 days of further extinction training. To validate Gq-DREADD activation of VP neurons, we injected mice with either CNO or Veh and immunostained for expression of the immediate-early gene product Fos 2 h afterward. All VP neurons, including VP_{GABA}, VP_{Glu}, and VP_{Penk} neurons, transduced with Gq-DREADD expressed elevated levels of Fos in response to CNO (Figures S1A–S1G; paired t tests, see figure legend for details). Comparing between cell types, CNO stimulation of VP_{Glu} neurons caused significantly more Fos activation in neighboring non-infected VP neurons (Figure S1H; expressed as percentage mCherry neurons, ANOVA $F_{[2,\ 13]} = 6.966$, $p = 0.009$; or expressed as percentage Fos, ANOVA $F_{[2,\ 13]} = 14.08$, $p = 5.6 \times 10^{-4}$), suggesting that VP_{Glu} neurons may directly innervate other VP neurons or indirectly activate the VP through an extended circuit mechanism. Compared with the other cell types, a significantly larger proportion of VP_{Penk} neurons were Fos activated by CNO (Figure S1I; ANOVA $F_{[2,\ 13]} = 13.42$, $p = 6.9 \times 10^{-4}$), indicating that this subpopulation may be more excitable or responsive to Gq pathway signaling than VP_{Glu} or VP_{GABA} neurons. All VP subpopulations (in viral-labeled and *in situ* hybridization-labeled tissue) were not randomly distributed and formed clusters based on k-nearest neighbor analysis (Figure S2; see figure legend for details).

All mouse strains acquired stable cocaine self-administration and extinguished responding in the absence of cocaine reward and drug-conditioned cues (see Figure 2C for wild-type mice transduced with non-floxed Gq DREADD in the VP and Figure S3 for Cre mice behavior). In the first experiment, wild-type mice were used and the VP was microinjected with AAV2-hSyn-hM3D-HA-mCitrine to transfect all neurons with Gq-DREADD regardless of subtype (Figure 2B). Stimulating all VP neurons augmented active nose-poke responses during extinction or cue-induced cocaine seeking (Figures 2D and 2E; extinction paired t test, $t_{[6]} = 2.89$, $p = 0.028$; cue seeking

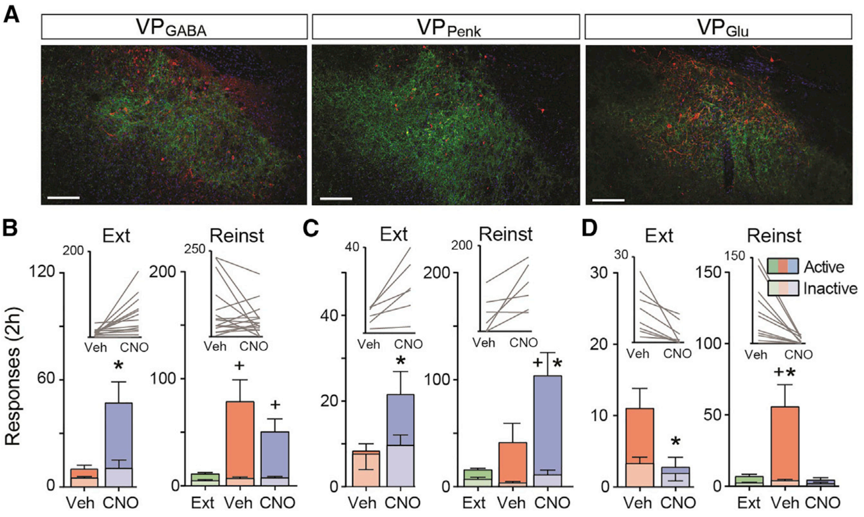

Figure 3. Selective Stimulation of VP Neuronal Subpopulations Differentially Affects Drug Seeking under Extinction Conditions and During Cue Seeking

(A) Representative micrographs showing Gq DREADD expression in the different subtypes of VP neurons. Substance P was used as a counterstain to delineate the VP. Scale bar, 150 μm.

(B–D) Outcome of selective chemogenetic stimulation of the three VP cell groups: VP_{GABA} (B), VP_{Penk} (C), and VP_{Glu} (D).

Data are presented as mean ± SEM. *p < 0.05 comparing between vehicle and CNO and +p < 0.05 comparing extinction and cue seeking.

repeated-measures [RM] ANOVA, $F_{[1.0,\ 6.2]} = 16.71$, p = 0.006). Inactive responses were also augmented, indicating non-selective behavioral activation (extinction paired t test, $t_{[6]} = 2.59$, p = 0.042; cue seeking RM ANOVA, $F_{[1.1,\ 6.8]} = 17.54$, p = 0.004). However, mice made significantly more active (previously cocaine rewarded) than inactive (unrewarded) responses during CNO facilitated cue-induced cocaine seeking indicating that stimulating the VP elicits drug seeking rather than non-specific motor activation (Figure 2E; two-way RM ANOVA interaction, cue seeking interaction $F_{[2,\ 12]} = 10.77$, p = 0.002). Active and inactive responding was not different during CNO-driven extinction responding, indicating potential nonspecific motor activation (Figure 2D; extinction interaction $F_{[1,\ 6]} = 4.13$, p = 0.186).

Gq-DREADD in VP_{GABA} Neurons

Given that the majority of VP neurons are GABAergic, we hypothesized that stimulating VP_{GABA} neurons would recapitulate the effects of global VP stimulation and transduced VP_{GABA} neurons with a Cre-dependent Gq DREADD in Vgat-IRES-Cre mice prior to cocaine self-administration training (Figure 3A; see Figure S3 for self-administration behavior). Indeed, when CNO was administered before a late extinction trial, the expected increase in active nose-poke responses was produced, although in contrast to nonspecific VP neuron stimulation, there was no increase in inactive responses (Figure 3B; active paired t test, $t_{[13]} = 3.02$, p = 0.010; inactive paired t test, $t_{[13]} = 1.25$, p = 0.235). Unexpectedly, although both CNO- and Veh-treated mice reinstated to cocaine-paired cues compared with extinction baseline, stimulating VP_{GABA} neurons with CNO did not potentiate reinstated cocaine seeking (Figure 3B; RM ANOVA, $F_{[1.6,\ 21.8]} = 7.25$, p = 0.006). There was no effect by CNO on inactive nose poking during cued cocaine seeking (Figure 3B; RM ANOVA, $F_{[1.6,\ 21.8]} = 1.52$, p = 0.240). Thus, although stimulating VP_{GABA} neurons produced a drug-seeking response in mice after extinction, it did not further potentiate drug seeking in the presence of conditioned drug cues.

Gq-DREADD in VP_{Penk} Neurons

We next expressed Gq DREADD in the GABAergic subpopulation of VP_{Penk} neurons using Penk-IRES-Cre mice that were trained to self-administer cocaine (Figure 3A; see Figure S3 for self-administration behavior and extinction). Following extinction learning, chemogenetic stimulation of VP_{Penk} augmented active nose-poke responding during both an extinction trial and during cue-induced cocaine seeking (Figure 3C; extinction paired t test, $t_{[5]} = 3.08$, p = 0.027; cue seeking RM ANOVA, $F_{[2,\ 10]} = 9.64$, p = 0.007). Stimulating VP_{Penk} neurons did not affect inactive responding during either the cue-induced cocaine seeking or extinction tests (Figure 3C; extinction paired t test, $t_{[5]} = 0.65$, p = 0.543; cue seeking RM ANOVA, $F_{[2,\ 10]} = 2.17$, p = 0.189). These data show that VP_{Penk} neurons constitute a subpopulation of VP_{GABA} that can facilitate cocaine seeking.

Gq-DREADD in VP_{Glu} Neurons

We also investigated the effect of stimulating VP_{Glu} neurons on cocaine seeking by expressing Gq-DREADD in Vglut2-IRES-Cre mice (Figure 3D; see Figure S3 for cocaine self-administration and extinction). Activating VP_{Glu} neurons reduced cocaine seeking during cocaine cue seeking (Figure 3D; RM-ANOVA, $F_{[2,\ 20]} = 11.95$, p = 0.006) and active nose poking during extinction (paired t test, $t_{[7]} = 3.18$, p = 0.016). Stimulating VP_{Glu} neurons did not affect inactive nose-poke responding (Figure 3D; extinction paired t test, $t_{[7]} = 1.00$, p = 0.351; cue seeking RM ANOVA, $F_{[2,\ 20]} = 2.19$, p = 0.180).

Single-Cell Ca^{2+} Imaging Reveals that Subpopulations of VP_{Glu} and VP_{Penk} Neurons Respond to Extinction and Cue-Induced Cocaine Seeking

We used a gradient index (GRIN) lens and miniature microscope strategy to quantify single-cell Ca^{2+} events in the different VP cell types. An AAV harboring a Ca^{2+} reporter (AAV1-hSyn-DIO-gCaMP6f) was microinjected into the VP of the different Cre mouse lines, and a GRIN lens (7.3 by 0.6 mm) was implanted above the microinjection site. Calcium-driven changes in fluorescence were visible within 3–4 weeks (Figures 4A and S4), and mice were implanted with jugular catheters and entered into the cocaine self-administration, extinction, and cued seeking protocol. Ca^{2+} events were quantified at three time points in the protocol, during the first day of extinction training (Ext1), when nose poking was extinguished to <40% of Ext1 after 10 d of extinction training (Ext10), and during cue-induced cocaine seeking (cue seeking). Mice underwent active

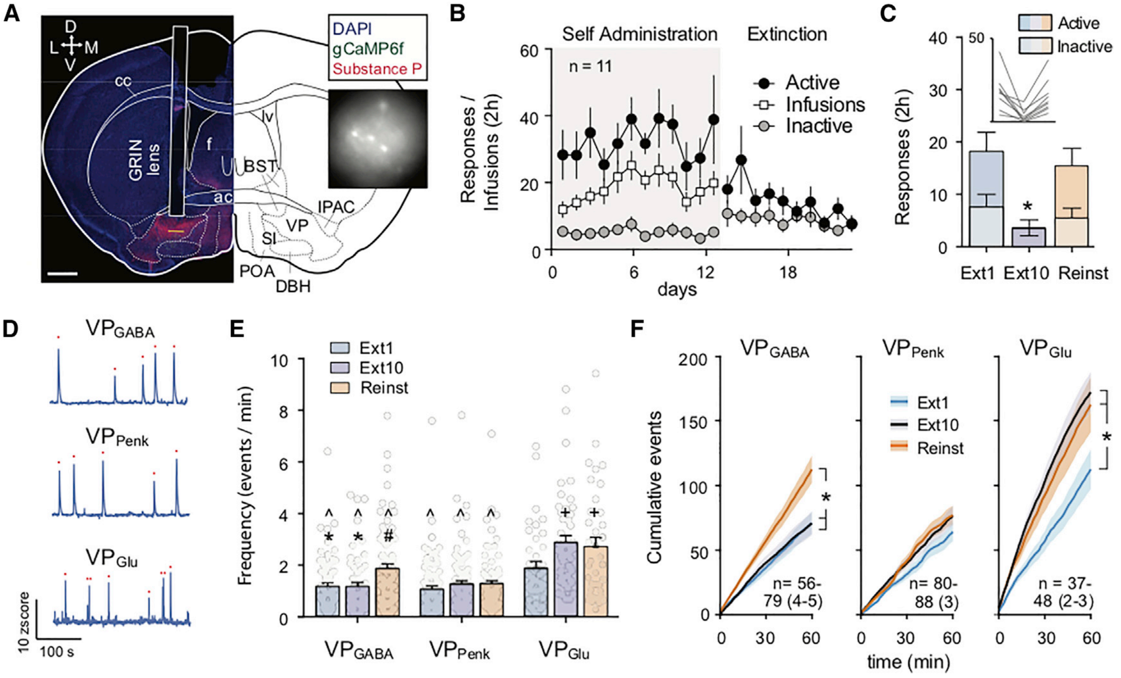

Figure 4. Miniscope Measurements of Single-Cell Ca^{2+} Spikes in VP during Extinction and Cue-Induced of Cocaine Seeking

(A) Representative micrograph of GRIN lens implantation site in a Vglut2-IRES-Cre mouse above VP to record from VP_{Glu} neurons expressing gCaMP6f. Inset shows example frame from miniature microscope video. BST, bed nucleus of the stria terminalis; cc, corpus callosum; DBH, diagonal horizontal band of Broca; f, fornix; ic, interior commissure; IPAC, interstitial nucleus of the posterior anterior commissure; lv, lateral ventricle; POA, preoptic area; SI, substantia innominata. Scale bar, 700 μm.

(B) Self-administration of cocaine (with dummy camera) and extinction training in all mice.

(C) Active and inactive nose pokes made in all mice used for Ca^{2+} imaging during Ext1, Ext10, and Reinst. * $p < 0.05$ comparing Ext10 to Ext1 and Reinst.

(D) Representative traces showing calcium events recorded from VP_{GABA}, VP_{Penk}, and VP_{Glu} neurons. Red dots indicate registered Ca^{2+} events.

(E) Comparisons between the average event rate across cell types and sessions. Comparisons between cell types revealed that VP_{Glu} neurons display a significantly higher Ca^{2+} event rate across all conditions compared with VP_{GABA} and VP_{Penk} neurons and that the VP_{GABA} Ca^{2+} event rate is elevated compared with VP_{Penk} during Reinst. Comparisons within cell types showed that extinction learning increases the frequency of Ca^{2+} events in VP_{Glu} (Ext1 versus Ext10 and Reinst), while Reinst selectively increases the Ca^{2+} event rate of VP_{GABA} (Reinst versus Ext1 and Ext10).

*$p < 0.05$ compared with cue seeking within cell group, +$p < 0.05$ compared with Ext1 within cell group, ^$p < 0.05$ compared with VP_{Glu} neurons in the same session, and #$p < 0.05$ compared with VP_{Penk} in the same session. (F) Cumulative spikes across the 2 h recording session. n = cell number per session over (mouse number). *$p < 0.05$ comparing between sessions. All data are presented as mean ± SEM.

nose-poke responding and cocaine infusions during self-administration when wearing a "dummy" miniature microscope (equal weight and dimensions compared with the recording device) for habituation (Figure 4B). Although the camera appeared to reduce responding on Ext1 (e.g., comparing between Figures 4B and 2C), responding during both Ext1 and Reinst was elevated compared with extinguished responding on Ext10 (Figure 4C; one-way RM ANOVA, $F_{[1.5,\ 11.8]} = 11.18$, $p = 0.003$).

We examined the effects of extinction training and cued cocaine seeking on the frequency and amplitude of Ca^{2+} events across cell types and sessions (Figure 4D). Because drug seeking is generally higher at the beginning of extinction and cued seeking sessions, and because of technical issues in some animals that prevented uninterrupted recordings during the entire 2 h session (e.g., tangling of the microscope data cable), analyses were restricted to the first 60 min of each session. We first compared the average number of calcium events between cell types and between conditions. VP_{Glu} neurons were more active than VP_{GABA} or VP_{Penk} neurons regardless of the session (Figure 4E; two-way ANOVA, interaction $F_{[4,\ 564]} = 2.879$, $p = 0.022$; main effects of cell type $F_{[2,\ 564]} = 37.13$, $p = 7.1 \times 10^{-16}$ and session $F_{[4,\ 564]} = 7.860$, $p = 4.3 \times 10^{-4}$). This increased number of Ca^{2+} events in VP_{Glu} neurons was most marked during Ext10, when the VP_{Glu} event rate (2.88 events/min) was >2-fold higher than that observed in VP_{Penk} or VP_{GABA} neurons. Comparing sessions within the VP_{Glu} population revealed that the VP_{Glu} Ca^{2+} event rate was increased after extinction learning had occurred (comparing Ext1 with Ext10 and Reinst). In VP_{GABA} neurons, the frequency of Ca^{2+} events was instead selectively increased during Reinst. By contrast, the frequency of VP_{Penk} Ca^{2+} events did not change across the sessions. Although the average calcium event rate across all recorded cells (1.58 events/min) was low compared with reported average basal electrical firing rates of VP neurons *in vivo* (282 spikes/min) (Richard et al., 2016), our findings are consistent with Ca^{2+} event rates reported for the nucleus accumbens (Francis et al., 2017) and lateral septum (Shin et al., 2018).

We next investigated at what point during these sessions changes in Ca^{2+} events would emerge within each cell type by plotting cumulative Ca^{2+} events over time (Figure 4F). This analysis confirmed the main changes in average Ca^{2+} activity described above. VP_{Glu} neurons were significantly more active after extinction training (during Ext10 and Reinst, compared with Ext1; two-way RM ANOVA, main effects of session $F_{[2,\ 120]} = 5.168$, $p = 0.007$; interaction $F_{[118,\ 7,080]} = 3.578$, $p = 1.9 \times 10^{-34}$; Figure 4F), and these differences in cumulative Ca^{2+} events between sessions became apparent after 22 min (Ext1 versus Ext10) and 27 min (Ext1 versus Reinst). Similarly, the increased Ca^{2+} event rate in VP_{GABA} neurons during Reinst (Figure 4F; two-way RM ANOVA, main effect of session $F_{[2,\ 197]} = 5.703$, $p = 0.004$; interaction $F_{[118,\ 11,623]} = 5.710$, $p = 6.1 \times 10^{-76}$) emerged at 27 min (Ext1 versus Reinst) and 30 min (Ext10 versus Reinst). Consistent with a lack of difference in the average VP_{Penk} Ca^{2+} event rate, analyzing cumulative Ca^{2+} events failed to identify an overall difference between sessions for VP_{Penk} neuron activity (Figure 4E; two-way ANOVA, effect of session $F_{[2,\ 247]} = 1.547$, $p = 0.215$). However, a significant interaction was found ($F_{[118,\ 14,573]} = 1.577$, $p = 7.1 \times 10^{-4}$; Figure 4F). Post hoc tests identified that this interaction was driven by significant ($p < 0.05$) differences between Ext1 and Reinst (minutes 37–38 and 54–58) and between Ext1 and Ext10 (minutes 55–56).

In contrast to the Ca^{2+} event frequency, the event amplitude did not change as a function of extinction learning or Reinst in any cell type (Figure S4B). However, the average Ca^{2+} event amplitude was higher in VP_{Penk} neurons than in VP_{Glu} and VP_{GABA} neurons (two-way ANOVA, main effect of cell-type $F_{[2,\ 564]} = 18.39$, $p < 0.001$; Figure S4B).

Although the increased occurrence of Ca^{2+} events in VP_{Glu} neurons following extinction learning is consistent with the inhibitory effect of this population identified in the Gq-DREADD study (see Figure 3D), to better understand the relationship between single neuron Ca^{2+} activity and cocaine seeking, we quantified activity across the epoch between the 10 s before and 20 s following an active nose poke in Ext1 and Reinst sessions. Analysis into the population activity of each cell type around nose pokes for cocaine revealed dynamics in Ca^{2+} population activity consistent with the distinct functional roles of each cell group during cocaine seeking (Figure 5). First, we investigated changes in Ca^{2+} activity around nose pokes in the different VP cell types expressed in absolute values to incorporate both increased and decreased responses as reported previously (Moorman and Aston-Jones, 2015; Figure 5A; see Figure S5 for separate response profiles for cells with increased or decreased activity). Overall, population activity in VP_{GABA} neurons was poorly organized around nose pokes (Figures 5A and 5B). A two-way ANOVA comparing the change in population activity around nose pokes (comparing activity 2 s before and 2 s after a nose poke) across Ext1 and Reinst identified a main effect of nose pokes on Ca^{2+} activity ($F_{[1,\ 142]} = 4.477$, $p = 0.036$; Figure 5B), but post hoc testing failed to identify significant changes in VP_{GABA} population activity around nose pokes during either Ext1 or Reinst. By contrast, VP_{Penk} and VP_{Glu} population activity was more consistently altered by nose pokes. A significant main effect of nose pokes on Ca^{2+} activity was found for VP_{Penk} population activity ($F_{[1,\ 160]} = 10.43$, $p = 0.002$), which post hoc tests confirmed was mediated by VP_{Penk} neuron activity around nose pokes during Reinst (Figure 5B). In addition, a significant main effect of nose pokes was observed for VP_{Glu} population activity ($F_{[1,\ 118]} = 14.76$, $p = 2.0 \times 10^{-4}$), and this effect was mediated by population activity around nose pokes during Ext1 (Figure 5B).

By organizing individual cells from the different sessions into heatmaps, Ca^{2+} activity dynamics were found to be carried by subpopulations with distinct activity patterns within each VP subgroup (Figure 5C). For each VP subgroup and recording session, Ca^{2+} activity of individual cells around nose pokes was classified as increased or decreased (Ca^{2+} activity up to 2 s after a nose-poke >2 SDs higher or lower than calcium activity 2 s prior to the nose poke; Figure 5D; see Figure S5A for example spatial cell maps and Figure S5B for separated temporal response patterns). During Ext1 and Reinst, VP_{GABA} and VP_{Glu} populations contained equivalent proportions of increased and decreased cells (Figure 5D). However, the proportion of activated and inhibited VP_{Penk} decreased significantly during Reinst compared with Ext1 (Figure 5D; chi-square = 9.82, $p = 0.007$). This reduction in the size of the population of VP_{Penk} neurons after extinction may have been due to a loss of neurons that responded to seeking in a context specific manner after that context had been extinguished.

DISCUSSION

Activity in VP neurons is necessary for motivated drug seeking in rodent models of drug addiction (Farrell et al., 2019; Mahler et al., 2014; McFarland and Kalivas, 2001; Prasad and McNally, 2016), but little is known regarding how activity in distinct VP cell types might differentially modulate drug seeking. We examined the two major cell types in a context- and cue-induced cocaine-seeking paradigm, VP_{GABA} neurons (73% of all neurons) and VP_{Glu} neurons (23%), as well as a subpopulation of VP_{GABA} neurons coexpressing proenkephalin (16%). We found that stimulating VP_{GABA} and VP_{Penk} neurons facilitated cocaine seeking and that cued seeking differentially activated these cells, by increasing in the number of Ca^{2+} events in VP_{GABA} and by changing Ca^{2+} population activity around nose pokes for VP_{Penk} neurons. Conversely, activating VP_{Glu} inhibited cocaine seeking, and VP_{Glu} Ca^{2+} events occurred more frequently following extinction learning. Moreover, VP_{Glu} population activity around nose-pokes was elevated selectively during early extinction when seeking responses no longer produced cocaine reward. Together, these data support a hypothesis that VP_{Glu} activity reduces motivation, that this process is recruited during extinction learning, and that VP_{GABA} and VP_{Penk} activity facilitates the motivation to seek cocaine in response to cocaine-associated cues.

In contrast to the association between chemogenetics-induced behavioral responses and Ca^{2+} activation of VP_{Penk} and VP_{Glu} neurons, chemogenetic VP_{GABA} stimulation did not alter cocaine seeking elicited by cocaine-conditioned cues, and Ca^{2+} population activity was not significantly organized around nose pokes during cued seeking. This more complex portrait of activity likely resulted from VP_{GABA} neurons' being a larger (∼73% of all VP neurons) and more heterogeneous cell

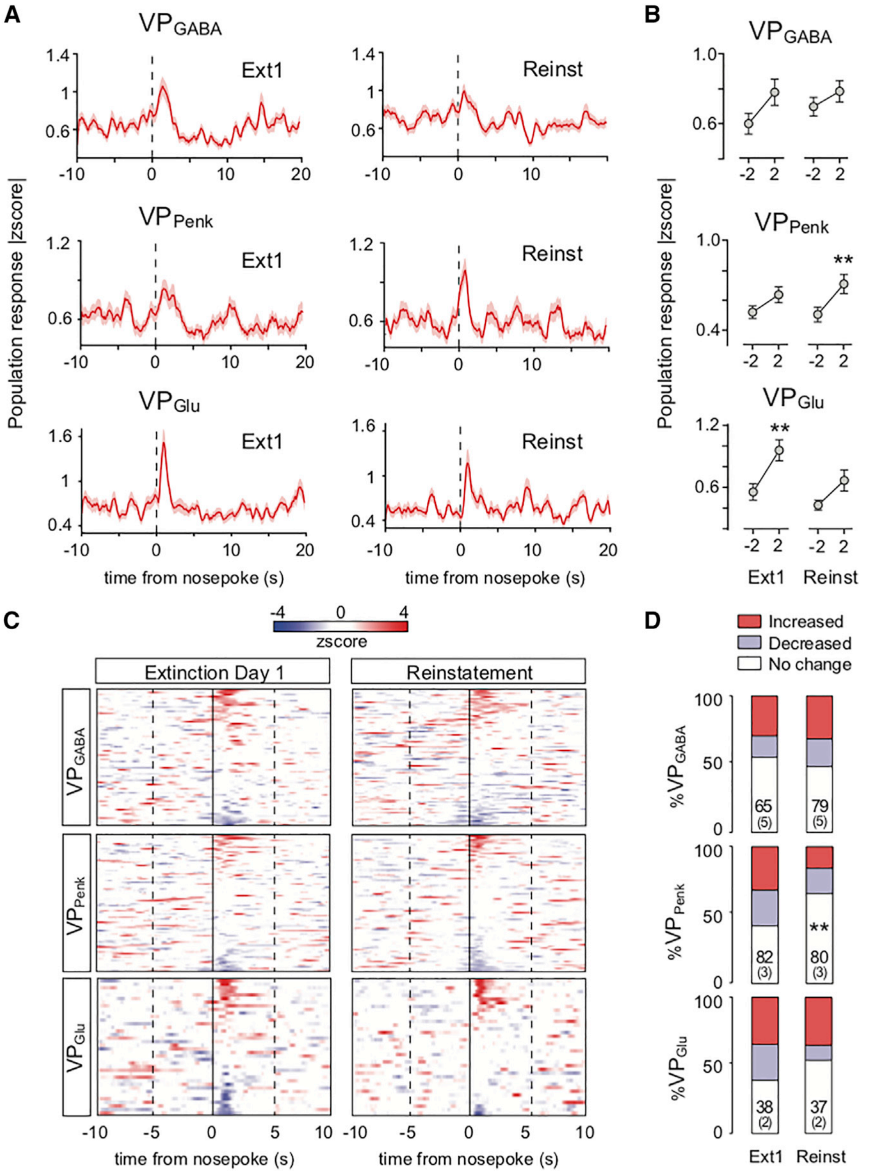

Figure 5. VP Subpopulations Show Distinct Patterns of Calcium Fluorescence Organized around Nose Pokes

(A) Peri-event histograms showing the normalized absolute change in population response magnitude for all VP neurons during Ext1 and Reinst before and after active nose pokes.

(B) Comparisons of the change in population activity between −2 s before and 2 s after a nose poke during Ext1 and Reinst. Changes in VP_{GABA} population calcium fluorescence were not significantly different around nose pokes during Ext1 or Reinst, VP_{Penk} population activity was altered around nose pokes during Reinst, and VP_{Glu} population activity changed around nose pokes during Ext1. **$p < 0.01$ comparing −2 s with 2 s around nose pokes.

(C) Overview of peri-event histograms showing distinct response patterns in each cell type during nose pokes, organized by activation or inhibition (increases in fluorescence are shown in red and decreases in fluorescence in blue).

(D) Population fractions of neurons with significantly (>2 SD) increased or decreased activity at 0–2 s following a nose poke. A smaller fraction of VP_{Penk} neurons was recruited during Reinst compared with Ext1.

n = cell number per session over (mouse number). Chi-square test. **$p < 0.01$ comparing fractions of activity patterns between Ext1 and Reinst. All data are presented as mean ± SEM.

group than VP_{Glu} and VP_{Penk} neurons. However, despite the lack of changes in Ca^{2+} activity around nose pokes in these cells, VP_{GABA} neurons strongly increased their overall Ca^{2+} event rate during cued seeking, indicating that these cells may mediate the overall increased motivated state associated with cue-induced drug seeking. Indeed, activation of VP_{GABA} cell bodies or VP_{GABA} projections to the VTA is rewarding (Faget et al., 2018), and GABA released from VP projections to the VTA is implicated in the reinstatement of cocaine seeking (Mahler et al., 2014). Thus, the increased activation of VP_{GABA} that we observed during reinstated cue seeking may have occluded further chemogenetic activation of these neurons, and this may explain the lack of VP_{GABA} chemogenetic potentiation of cocaine seeking initiated by cocaine-associated cues. Moreover, given that cue seeking strongly increased the Ca^{2+} event rate in VP_{GABA} neurons, and only subtle increases were observed in VP_{Penk} neurons, drug seeking during cue seeking likely requires non-enkephalin VP_{GABA} neurons, for instance those expressing parvalbumin (Knowland et al., 2017).

Although our chemogenetic manipulations identified distinct contributions of VP subpopulations on drug seeking during extinction and cocaine cue seeking, it should be noted that these manipulations only demonstrate that activation of these cell populations is sufficient for driving or inhibiting drug seeking. Future studies are required to verify the necessity of activity in these cell groups for drug seeking and for extinction learning. To date, multiple studies have demonstrated that VP activity is required for the reinstated drug seeking (Farrell et al., 2019; Mahler et al., 2014; McFarland and Kalivas, 2001; Prasad and McNally, 2016; Rogers et al., 2008), and given that reinstated drug seeking requires a GABA-mediated disinhibition of VTA dopamine neurons and functional VP-VTA projections, cued drug seeking likely depends on VP_{GABA} activation (Mahler et al., 2014). It should also be noted that although we observed an overall consistency between the effects of chemogenetic stimulation of VP cell types and their activation patterns revealed by Ca^{2+} imaging, chemogenetic manipulations act on a slow timescale. More specific brief optogenetic inhibition of VP neurons during cue presentations disrupts cue-reward processing (Richard et al., 2016), suggesting

that VP neurons are required for integrating reward predictive information to drive reward seeking. Future experiments using similar temporally specific activation or inhibition strategies in VP subpopulations during operant drug-seeking responses could further elucidate a more precise role for these cells and their projections during drug seeking.

We observed marked changes in the activation of VP cell types over the course of extinction learning and during cue-induced reinstatement of cocaine seeking. Indeed, VP_{Glu} activity across the session, measured by the frequency of Ca^{2+} events, increased following extinction learning. Because of the inhibitory role that we observed for VP_{Glu} neuron activation, we ascribe this change to the effects of extinction learning, whereby animals learn to inhibit their responding in the extinguished context. However, whether the inhibitory role of this population changes as a direct result of extinction learning, as has been shown for other brain structures (Peters et al., 2009), requires further investigation. For instance, altered VP_{Glu} activity following extinction learning may be mediated by changes in excitatory versus inhibitory synaptic inputs onto these neurons (Knowland et al., 2017; McDaid et al., 2005). Similar synaptic changes may account for the increased activation of VP_{GABA} neurons during cued cocaine seeking. Indeed, previous work from our lab and others identified a selective loss of inhibitory inputs from D2-MSNs onto VP neurons after withdrawal from cocaine self-administration (Creed et al., 2016; Heinsbroek et al., 2017; Kupchik et al., 2014). Reduced GABA release in the VP occurs during reinstated cocaine seeking, and this process likely disinhibits VP neurons (Tang et al., 2005). Future studies should address whether this disinhibition is specific to VP_{GABA} neurons and whether this is the underlying mechanism for the observed increase in VP_{GABA} neurons during cued cocaine seeking in the present study.

In addition to changes in the activation of VP_{Glu} and VP_{GABA} neurons, we also observed a subtle increase in the number of Ca^{2+} events in VP_{Penk} neurons over the course of late extinction and cued seeking sessions. The aforementioned reduced functioning of inhibitory synapses onto VP neurons after cocaine self-administration is mediated by an increased enkephalinergic tone onto presynaptic μ opioid receptors expressed on GABAergic afferents from the nucleus accumbens (Heinsbroek et al., 2017; Kupchik et al., 2014; Tang et al., 2005). This increased enkephalinergic tone may be produced by an increase in VP_{Penk} activity after cocaine withdrawal. We also observed a reduction in the number of VP_{Penk} neurons that were modulated by nose pokes for cocaine between early extinction and cued cocaine seeking. During early extinction, the VP_{Penk} population responding to nose pokes likely included neurons that respond to contextual cues (i.e., the self-administration chamber) and discrete (non-extinguished) cues. Following extinction, the context-coding cells may no longer have been reflected in VP_{Penk} Ca^{2+} events (i.e., because the context was extinguished), resulting in a reduced number of cells recruited by nose pokes during cue seeking. It is interesting to note that despite a reduction in the size of the VP_{Penk} population recruited by nose pokes, overall VP_{Penk} population responses were recruited by nose pokes only during cue seeking. This may be explained by a sharpening of the population response to nose pokes when only cue-activated VP_{Penk} neurons were responsive, but this hypothesis requires further testing in future studies. It also remains possible that the differences observed between early extinction and cue seeking in VP_{Penk} neurons are mediated by subsets of VP_{Penk} neurons defined by distinct projection targets. In line with this idea, enkephalin-containing globus pallidus neurons project to the striatum and encode a stop signal for ongoing movement patterns (Mallet et al., 2016). However, VP enkephalin neurons also project to the VTA, where the activation of μ opioid receptors by enkephalin is linked to the activation of dopamine neurons (Johnson and North, 1992; Kalivas et al., 1993) and behavioral activation (Kalivas and Richardson-Carlson, 1986; Stewart, 1984).

Indeed, an important finding across VP_{GABA}, VP_{Penk}, and VP_{Glu} neurons is that although stimulating VP subpopulations resulted in consistent behavioral responses, measures of Ca^{2+} activity revealed substantial heterogeneity within each cell group. These distinct subpopulations and patterns of activity may arise from a number of factors. Although mice were well trained, operant responding contains nuanced behavioral sequences associated with nose poking that may be encoded in the VP. Also, different neurons have distinct axonal projections or afferent inputs that differentially undergo adaptations in response to operant training or cocaine use (Faget et al., 2018; Heinsbroek et al., 2017; Tooley et al., 2018). For example, there is differential input from D1- and D2-MSNs (Creed et al., 2016; Kupchik et al., 2015; Figure 1) to the various VP neuronal subpopulations, which project to different downstream nuclei. Also, VP_{Glu} neurons densely innervate the lHb, midline thalamus, lateral hypothalamus, and ventral mesencephalon, while VP_{Penk} neurons project to the ventral mesencephalon and likely ventral striatum (Faget et al., 2018; Kalivas et al., 1993; Mallet et al., 2012; Tooley et al., 2018), and subpopulations containing different connectivity may contribute differentially to behavior. Consistent with this possibility, optogenetic stimulation of VP_{GABA} cell bodies or projections to the VTA produces place preference, whereas stimulating VP_{GABA} habenula projections does not elicit any behavioral effects (Faget et al., 2018). Given that VP_{Glu} and VP_{GABA} neurons both project to the lHb and VTA, the distinct contributions of these pathways to drug seeking warrants further investigation (Barker et al., 2017; Faget et al., 2018).

In conclusion, we have characterized cell types in the VP with opposing effects on cocaine seeking. VP_{GABA} and VP_{Penk} activity facilitates, while VP_{Glu} activity inhibits, cocaine seeking. These behavioral roles were largely consistent with measures of single cell Ca^{2+} activity, in which a subpopulation of VP_{Penk} neurons was associated with cued nose poking, and VP_{GABA} Ca^{2+} events were increased, while VP_{Glu} Ca^{2+} events were increased after extinction training and during cocaine cue seeking. Our data constitute a first step toward disentangling how the accumbens to VP subcircuits regulate cocaine seeking and are a harbinger of future studies to examine how ensembles of VP subpopulations function in concert to regulate drug seeking and relapse.

STAR★METHODS

Detailed methods are provided in the online version of this paper and include the following:

- KEY RESOURCES TABLE
- LEAD CONTACT AND MATERIALS AVAILABILITY

- EXPERIMENTAL MODEL AND SUBJECT DETAILS
  - Animals
- METHOD DETAILS
  - Catheter surgery and Chemogenetics
  - Self-administration training and testing
  - *In vivo* Ca^{2+} imaging
  - Monosynaptic retrograde rabies tracing
  - Histology
  - *In situ* hybridization
- QUANTIFICATION AND STATISTICAL ANALYSIS
  - Ca^{2+} imaging data analysis
  - Histology clustering analysis
  - Statistics
- DATA AND CODE AVAILABILITY

SUPPLEMENTAL INFORMATION

Supplemental Information can be found online at https://doi.org/10.1016/j.celrep.2020.01.023.

ACKNOWLEDGMENTS

We thank Dr. Tom Jhou for original breeder mice used to establish Vgat-IRES-Cre and Penk-IRES-Cre colonies; Dr. Jan Boddaert and Thibaut Pardo-Garcia for assistance with Fos analyses; Drs. Constanza Garcia-Keller and Davide Amato for assistance with Ca^{2+} imaging; and Savanna Gonzalez, Nicholas Fayette, Paco Herson, and members of the Kalivas lab for edits to the manuscript and discussion of experiments. In addition, we would like to thank Dr. Patrick Mulholland and the Shared Confocal Core for microscopy use and guidance. This work was supported by National Institutes of Health grants DA00396, DA012513, DA046373, DP5 OD026407, and S10OD021532.

AUTHOR CONTRIBUTIONS

Conceptualization, J.A.H. and P.W.K.; Methodology, J.A.H. and A.A.; Investigation, J.A.H., A.-C.B., E.D., A.A., and R.M.C.; Resources, C.W.C; Writing – Original Draft, J.A.H. and P.W.K.; Writing – Review & Editing, J.A.H., A.-C.B., R.M.C., C.W.C., and P.W.K.

DECLARATION OF INTERESTS

The authors declare no competing interests.

Received: September 10, 2019
Revised: November 11, 2019
Accepted: January 7, 2020
Published: February 11, 2020

REFERENCES

Barker, D.J., Miranda-Barrientos, J., Zhang, S., Root, D.H., Wang, H.L., Liu, B., Calipari, E.S., and Morales, M. (2017). Lateral preoptic control of the lateral habenula through convergent glutamate and GABA transmission. Cell Rep. *21*, 1757–1769.

Beier, K.T., Kim, C.K., Hoerbelt, P., Hung, L.W., Heifets, B.D., DeLoach, K.E., Mosca, T.J., Neuner, S., Deisseroth, K., Luo, L., and Malenka, R.C. (2017). Rabies screen reveals GPe control of cocaine-triggered plasticity. Nature *549*, 345–350.

Creed, M., Ntamati, N.R., Chandra, R., Lobo, M.K., and Lüscher, C. (2016). Convergence of reinforcing and anhedonic cocaine effects in the ventral pallidum. Neuron *92*, 214–226.

Dodson, P.D., Larvin, J.T., Duffell, J.M., Garas, F.N., Doig, N.M., Kessaris, N., Duguid, I.C., Bogacz, R., Butt, S.J., and Magill, P.J. (2015). Distinct developmental origins manifest in the specialized encoding of movement by adult neurons of the external globus pallidus. Neuron *86*, 501–513.

Faget, L., Zell, V., Souter, E., McPherson, A., Ressler, R., Gutierrez-Reed, N., Yoo, J.H., Dulcis, D., and Hnasko, T.S. (2018). Opponent control of behavioral reinforcement by inhibitory and excitatory projections from the ventral pallidum. Nat. Commun. *9*, 849.

Farrell, M.R., Ruiz, C.M., Castillo, E., Faget, L., Khanbijian, C., Liu, S., Schoch, H., Rojas, G., Huerta, M.Y., Hnasko, T.S., et al. (2019). Ventral pallidum is essential for cocaine relapse after voluntary abstinence in rats. Neuropsychopharmacology *44*, 2174–2185.

Francis, T.C., Chandra, R., Gaynor, A., Konkalmatt, P., Metzbower, S.R., Evans, B., Engeln, M., Blanpied, T.A., and Lobo, M.K. (2017). Molecular basis of dendritic atrophy and activity in stress susceptibility. Mol. Psychiatry *22*, 1512–1519.

Geisler, S., Derst, C., Veh, R.W., and Zahm, D.S. (2007). Glutamatergic afferents of the ventral tegmental area in the rat. J. Neurosci. *27*, 5730–5743.

Gerfen, C.R., Paletzki, R., and Heintz, N. (2013). GENSAT BAC cre-recombinase driver lines to study the functional organization of cerebral cortical and basal ganglia circuits. Neuron *80*, 1368–1383.

Gong, S., Zheng, C., Doughty, M.L., Losos, K., Didkovsky, N., Schambra, U.B., Nowak, N.J., Joyner, A., Leblanc, G., Hatten, M.E., and Heintz, N. (2003). A gene expression atlas of the central nervous system based on bacterial artificial chromosomes. Nature *425*, 917–925.

Harris, J.A., Hirokawa, K.E., Sorensen, S.A., Gu, H., Mills, M., Ng, L.L., Bohn, P., Mortrud, M., Ouellette, B., Kidney, J., et al. (2014). Anatomical characterization of Cre driver mice for neural circuit mapping and manipulation. Front. Neural Circuits *8*, 76.

Heimer, L. (1975). The subcortical projections of the allocortex: similarities in the neural associations of the hippocampus, the piriform cortex, and the neocortex. Golgi Centennial Symposium Proceedings, pp. 177–193.

Heimer, L., Zahm, D.S., Churchill, L., Kalivas, P.W., and Wohltmann, C. (1991). Specificity in the projection patterns of accumbal core and shell in the rat. Neuroscience *41*, 89–125.

Heinsbroek, J.A., Neuhofer, D.N., Griffin, W.C., 3rd, Siegel, G.S., Bobadilla, A.C., Kupchik, Y.M., and Kalivas, P.W. (2017). Loss of plasticity in the D2-accumbens pallidal pathway promotes cocaine seeking. J. Neurosci. *37*, 757–767.

Hur, E.E., and Zaborszky, L. (2005). Vglut2 afferents to the medial prefrontal and primary somatosensory cortices: a combined retrograde tracing in situ hybridization study [corrected]. J. Comp. Neurol. *483*, 351–373.

Jennings, J.H., Ung, R.L., Resendez, S.L., Stamatakis, A.M., Taylor, J.G., Huang, J., Veleta, K., Kantak, P.A., Aita, M., Shilling-Scrivo, K., et al. (2015). Visualizing hypothalamic network dynamics for appetitive and consummatory behaviors. Cell *160*, 516–527.

Johnson, S.W., and North, R.A. (1992). Opioids excite dopamine neurons by hyperpolarization of local interneurons. J. Neurosci. *12*, 483–488.

Kalivas, P.W., and Richardson-Carlson, R. (1986). Endogenous enkephalin modulation of dopamine neurons in ventral tegmental area. Am. J. Physiol. *251*, R243–R249.

Kalivas, P.W., Churchill, L., and Klitenick, M.A. (1993). GABA and enkephalin projection from the nucleus accumbens and ventral pallidum to the ventral tegmental area. Neuroscience *57*, 1047–1060.

Knowland, D., Lilascharoen, V., Pacia, C.P., Shin, S., Wang, E.H., and Lim, B.K. (2017). Distinct ventral pallidal neural populations mediate separate symptoms of depression. Cell *170*, 284–297.e18.

Kupchik, Y.M., Scofield, M.D., Rice, K.C., Cheng, K., Roques, B.P., and Kalivas, P.W. (2014). Cocaine dysregulates opioid gating of GABA neurotransmission in the ventral pallidum. J. Neurosci. *34*, 1057–1066.

Kupchik, Y.M., Brown, R.M., Heinsbroek, J.A., Lobo, M.K., Schwartz, D.J., and Kalivas, P.W. (2015). Coding the direct/indirect pathways by D1 and D2 receptors is not valid for accumbens projections. Nat. Neurosci. *18*, 1230–1232.

Madisen, L., Zwingman, T.A., Sunkin, S.M., Oh, S.W., Zariwala, H.A., Gu, H., Ng, L.L., Palmiter, R.D., Hawrylycz, M.J., Jones, A.R., et al. (2010). A robust and high-throughput Cre reporting and characterization system for the whole mouse brain. Nat. Neurosci. *13*, 133–140.

Mahler, S.V., Vazey, E.M., Beckley, J.T., Keistler, C.R., McGlinchey, E.M., Kaufling, J., Wilson, S.P., Deisseroth, K., Woodward, J.J., and Aston-Jones, G. (2014). Designer receptors show role for ventral pallidum input to ventral tegmental area in cocaine seeking. Nat. Neurosci. *17*, 577–585.

Mallet, N., Micklem, B.R., Henny, P., Brown, M.T., Williams, C., Bolam, J.P., Nakamura, K.C., and Magill, P.J. (2012). Dichotomous organization of the external globus pallidus. Neuron *74*, 1075–1086.

Mallet, N., Schmidt, R., Leventhal, D., Chen, F., Amer, N., Boraud, T., and Berke, J.D. (2016). Arkypallidal cells send a stop signal to striatum. Neuron *89*, 308–316.

Matsui, A., and Alvarez, V.A. (2018). Cocaine inhibition of synaptic transmission in the ventral pallidum is pathway-specific and mediated by serotonin. Cell Rep. *23*, 3852–3863.

McDaid, J., Dallimore, J.E., Mackie, A.R., Mickiewicz, A.L., and Napier, T.C. (2005). Cross-sensitization to morphine in cocaine-sensitized rats: behavioral assessments correlate with enhanced responding of ventral pallidal neurons to morphine and glutamate, with diminished effects of GABA. J. Pharmacol. Exp. Ther. *313*, 1182–1193.

McFarland, K., and Kalivas, P.W. (2001). The circuitry mediating cocaine-induced reinstatement of drug-seeking behavior. J. Neurosci. *21*, 8655–8663.

Moorman, D.E., and Aston-Jones, G. (2015). Prefrontal neurons encode context-based response execution and inhibition in reward seeking and extinction. Proc. Natl. Acad. Sci. U S A *112*, 9472–9477.

Ottenheimer, D., Richard, J.M., and Janak, P.H. (2018). Ventral pallidum encodes relative reward value earlier and more robustly than nucleus accumbens. Nat. Commun. *9*, 4350.

Pardo-Garcia, T.R., Garcia-Keller, C., Penaloza, T., Richie, C.T., Pickel, J., Hope, B.T., Harvey, B.K., Kalivas, P.W., and Heinsbroek, J.A. (2019). Ventral pallidum is the primary target for accumbens D1 projections driving cocaine seeking. J. Neurosci. *39*, 2041–2051.

Peters, J., Kalivas, P.W., and Quirk, G.J. (2009). Extinction circuits for fear and addiction overlap in prefrontal cortex. Learn. Mem. *16*, 279–288.

Prasad, A.A., and McNally, G.P. (2016). Ventral pallidum output pathways in context-induced reinstatement of alcohol seeking. J. Neurosci. *36*, 11716–11726.

Richard, J.M., Ambroggi, F., Janak, P.H., and Fields, H.L. (2016). Ventral pallidum neurons encode incentive value and promote cue-elicited instrumental actions. Neuron *90*, 1165–1173.

Roberts-Wolfe, D., Bobadilla, A.C., Heinsbroek, J.A., Neuhofer, D., and Kalivas, P.W. (2018). Drug refraining and seeking potentiate synapses on distinct populations of accumbens medium spiny neurons. J. Neurosci. *38*, 7100–7107.

Rogers, J.L., Ghee, S., and See, R.E. (2008). The neural circuitry underlying reinstatement of heroin-seeking behavior in an animal model of relapse. Neuroscience *151*, 579–588.

Root, D.H., Melendez, R.I., Zaborszky, L., and Napier, T.C. (2015). The ventral pallidum: Subregion-specific functional anatomy and roles in motivated behaviors. Prog. Neurobiol. *130*, 29–70.

Root, D.H., Zhang, S., Barker, D.J., Miranda-Barrientos, J., Liu, B., Wang, H.L., and Morales, M. (2018). Selective brain distribution and distinctive synaptic architecture of dual glutamatergic-GABAergic neurons. Cell Rep. *23*, 3465–3479.

Shin, S., Pribiag, H., Lilascharoen, V., Knowland, D., Wang, X.Y., and Lim, B.K. (2018). Drd3 signaling in the lateral septum mediates early life stress-induced social dysfunction. Neuron *97*, 195–208.e6.

Smith, A.C.W., Scofield, M.D., Heinsbroek, J.A., Gipson, C.D., Neuhofer, D., Roberts-Wolfe, D.J., Spencer, S., Garcia-Keller, C., Stankeviciute, N.M., Smith, R.J., et al. (2017). Accumbens nNOS interneurons regulate cocaine relapse. J. Neurosci. *37*, 742–756.

Stefanik, M.T., Kupchik, Y.M., Brown, R.M., and Kalivas, P.W. (2013). Optogenetic evidence that pallidal projections, not nigral projections, from the nucleus accumbens core are necessary for reinstating cocaine seeking. J. Neurosci. *33*, 13654–13662.

Stewart, J. (1984). Reinstatement of heroin and cocaine self-administration behavior in the rat by intracerebral application of morphine in the ventral tegmental area. Pharmacol. Biochem. Behav. *20*, 917–923.

Tang, X.C., McFarland, K., Cagle, S., and Kalivas, P.W. (2005). Cocaine-induced reinstatement requires endogenous stimulation of mu-opioid receptors in the ventral pallidum. J. Neurosci. *25*, 4512–4520.

Tooley, J., Marconi, L., Alipio, J.B., Matikainen-Ankney, B., Georgiou, P., Kravitz, A.V., and Creed, M.C. (2018). Glutamatergic ventral pallidal neurons modulate activity of the habenula-tegmental circuitry and constrain reward seeking. Biol. Psychiatry *83*, 1012–1023.

Vong, L., Ye, C., Yang, Z., Choi, B., Chua, S., Jr., and Lowell, B.B. (2011). Leptin action on GABAergic neurons prevents obesity and reduces inhibitory tone to POMC neurons. Neuron *71*, 142–154.

Wallace, M.L., Saunders, A., Huang, K.W., Philson, A.C., Goldman, M., Macosko, E.Z., McCarroll, S.A., and Sabatini, B.L. (2017). Genetically distinct parallel pathways in the entopeduncular nucleus for limbic and sensorimotor output of the basal ganglia. Neuron *94*, 138–152.e5.

Zhu, C., Yao, Y., Xiong, Y., Cheng, M., Chen, J., Zhao, R., Liao, F., Shi, R., and Song, S. (2017). Somatostatin neurons in the basal forebrain promote high-calorie food intake. Cell Rep. *20*, 112–123.

STAR★METHODS

KEY RESOURCES TABLE

REAGENT or RESOURCE	SOURCE	IDENTIFIER
Antibodies		
Rabbit anti-Substance P	Immunostar	#20064; RRID:AB_572266
Rabbit anti-dsRed	Clontech	#632496; RRID:AB_10013483
Rabbit anti-ser32-phospho-cFos	Cell signaling	#5348; RRID:AB_10557109
Chicken anti-mCherry	LifeSpan	LS-C204825, RRID:AB_2716246
Chicken anti-GFP	Abcam	ab13970, RRID:AB_300798
Goat anti-Rabbit Alexa 488	Thermo Fisher	A-11008, RRID:AB_143165
Goat anti-Rabbit Alexa 594	Thermo Fisher	A-11012, RRID:AB_2534079
Goat anti-Chicken Alexa 488	Thermo Fisher	A-11039, RRID:AB_2534096
Goat anti-Chicken Alexa 594	Thermo Fisher	A-11042, RRID:AB_2534099
Bacterial and Virus Strains		
AAV1-hSyn-DIO-gCaMP6f	Addgene	100833-AAV1
AAV2-hSyn-DIO-hM3D-mCherry	Addgene	44361-AAV2
AAV2-hSyn-hM3D-HA-mCitrine	UNC Vector Core	N/A
AAV8-CA-DIO-RG	UNC Vector Core	N/A
AAV2-Ef1a-DIO-GT	Salk Vector Core	N/A
EnvA- SADΔG-B19-mCherry	Salk Vector Core	N/A
Chemicals, Peptides and Recombinant Proteins		
Cocaine Hydrochloride	NIDA	N/A
Clozapine-N-oxide	Abcam	Cat# ab141704
DAPI	ACDbio	Cat# 320858
RNAscope® Multiplex Fluorescent Detection Kit v2	ACDbio	Cat# 323110
Hydrogen Peroxide	ACDbio	Cat# 322330
Opal 520	Perkin Elmer	Cat# FP1487001KT
Opal 570	Perkin Elmer	Cat# FP1488001KT
Opal 690	Perkin Elmer	Cat# FP1497001KT
Prolong Gold	Thermo Fisher	Cat# P36934
Experimental Models: Organisms/Strains		
Vglut2-IRES-Cre	Jackson Lab	RRID:IMSR_JAX:028863
Vgat-IRES-Cre	Jackson Lab	RRID:IMSR_JAX:028862
Penk-IRES2-Cre	Jackson Lab	RRID:IMSR_JAX:025112
C57BL/6J	Jackson Lab	RRID:IMSR_JAX:000664
Drd2-eGFP	GENSAT	RRID:MMRRC_000230-UNC
Software and Algorithms		
MATLAB	Mathworks	RRID:SCR_001622
Fiji ImageJ	NIH	RRID:SCR_002285
Imaris	Bitplane	RRID:SCR_007370
Prism	Graphpad	RRID:SCR_005375
nVista	Inscopix	RRID:SCR_017407
Mosaic	Inscopix	RRID:SCR_017408

LEAD CONTACT AND MATERIALS AVAILABILITY

This study did not generate new unique reagents. Further information and requests for resources and reagents should be directed to and will be fulfilled by the Lead Contact, Peter Kalivas (kalivasp@musc.edu)

EXPERIMENTAL MODEL AND SUBJECT DETAILS

Animals

Male and female transgenic mice were bred at Medical University of South Carolina (MUSC) and every other generation new mice were introduced into the colonies to prevent inbreeding (Vglut2-IRES-Cre, Vgat-IRES-Cre, Penk-IRES-Cre, JAX laboratories; Drd2-eGFP mice gifted by the GENSET program at Rockefeller University) (Gerfen et al., 2013; Gong et al., 2003; Harris et al., 2014; Madisen et al., 2010; Vong et al., 2011) For anatomical tracing and electrophysiological studies Vglut2-IRES-Cre, Vgat-IRES-Cre and Penk-IRES-Cre were crossed with Drd2-eGFP lines. All mice were housed on a reverse day light cycle, and provided with access to food and water *ad libitum* until the start of experiments. All experiments were conducted in accordance with the National Institute of Health's Guidelines for the care and use of laboratory animals and approved by the Institutional Animal Care and Use Committee at MUSC.

METHOD DETAILS

Catheter surgery and Chemogenetics

Mice were implanted with a chronic jugular vein catheter as described previously (Heinsbroek et al., 2017; Smith et al., 2017) and infused (~200 nL over 10 min) into the VP (AP: 0.4, ML: 1.4, DV: −5) with the following AAV vectors: AAV2-hSyn-hM3D-HA-mCitrine (University of North Carolina vector core); AAV2-hSyn-DIO-hM3D-mCherry (Addgene). Cre dependent vectors were validated in wild-type mice to test for non-specific expression, and virus was given at least 3 weeks of expression before behavioral tests were conducted.

Self-administration training and testing

Three to four days after surgery, mice were food deprived overnight and trained to self-administer cocaine (0.75 mg/kg/infusion; FR1 schedule of reinforcement) for 12 days, during which operant responses for cocaine were paired with the presentation of a compound cue (tone + light). Throughout self-administration catheter patency was assessed periodically using the intravenous infusion of the short acting barbiturate Brevital. Afterward, mice underwent 7 days of home cage abstinence, followed by at least 10 days of extinction training with neither cocaine nor cocaine-associated cues present, followed by cue seeking tests (see: Figure 2A for experimental timeline). During cue-induced cue seeking tests, conditioned cues were returned to every active operant response to stimulate cocaine seeking. During extinction tests, cue seeking was induced by chemogenetic stimulation of the different populations of VP neurons during a regular extinction session. Across all studies approximately 70% of the mice successfully completed self-administration and extinction training.

In vivo Ca^{2+} imaging

Mice were injected with the Cre dependent genetically encoded Ca^{2+} indicator gCaMP6f (AAV1-hSyn-DIO-gCaMP6f; titer: ~$1*10^{13}$ GC/ml; University of Pennsylvania vector core and Addgene) into the VP, followed by the implantation of a GRIN lens (7.3 mm by 0.6 mm; Inscopix) above the VP. Mice were allowed to recover for 3 weeks, and underwent a second surgery during which a base-plate was installed for miniature microscope attachment. Videos of Ca^{2+} mediated fluorescence changes in individual neurons were acquired using a miniature microscope system and acquisition software (nVista, Inscopix). Videos were recorded at a 15 Hz framerate under constant low light illumination < 1 mW. Data files were decompressed, downsampled by a special factor of four, motion corrected using a rigid body translation algorithm (TurboReg), and ΔF/F normalized (Mosaic, Inscopix). Afterward, temporal and spatial components were extracted using principal/independent component analysis (PCA/ICA). The quality of individual temporal and spatial components was examined manually for each cell using Mosaic software (Inscopix). Changes in Ca^{2+} fluorescence around behaviorally meaningful events (nose-pokes for cocaine) and the frequency and amplitude of Ca^{2+} transients (Ca^{2+} events) were analyzed separately. Ca^{2+} events were identified using a peak-finder algorithm (Mosaic, Inscopix) and frequency (spikes/min) and amplitude (a.u.) data were processed using custom-written code in MATLAB (Mathworks).

Monosynaptic retrograde rabies tracing

For cell-type specific monosynaptic retrograde rabies tracing, D2-eGFP x Cre recombinase expressing double transgenic mice were injected with a cocktail of Cre dependent helper viruses mixed in 1:1 ratio (AAV2-Ef1a-DIO-GT, titer: $4.4*10^{12}$ GC/ml, Salk vector core; AAV8-CA-DIO-RG, titer: $2.5*10^{12}$ GC/ml, UNC Vector core). Three weeks later mice were injected with G-Deleted EnvA pseudotyped replication deficient Rabies SADΔG-B19-mCherry (Salk vector core, titer: $2.7*10^{8}$ TU/ml). Ten days after rabies injection, animals were perfused for histology.

Histology

Mice were deeply anesthetized with isoflurane, and transcardially perfused with ice-cold saline followed by 10% formalin. Immunostaining was performed using primary antibodies against GFP (1:500; Abcam ab13970; RRID:AB_300798), mCherry (1:20k; LS-C204825; RRID:AB_2716246), dsRed (1:1000; Clontech #632496; RRID:AB_10013483), substance P (1:1000, Immunostar #20064, RRID:AB_572266), and Ser32-phospho-cFos (1:2000, Cell signaling #5348; RRID:AB_10557109), as well as Alexa-conjugated

secondary antibodies (Invitrogen, 1:500). Cell counting and co-localization studies were performed using the cell counting plugin in ImageJ (NIH). For immunohistochemical validation of DREADD function, mice were injected with CNO or vehicle 2 h prior to perfusion.

In situ hybridization

Wild-type C57BL/6J mice were euthanized, and brains were rapidly extracted and flash frozen in isopentane solution on dry ice. Brains slices (15 μm) were stained for mRNA expression using the RNAscope *in situ* hybridization protocol (ACDbio) based on manufacturer recommendations, without epitope retrieval or protease pretreatment steps. Images were taken using a Zeiss LSM880 confocal microscope at 40x and cell counts and co-localization analyses were performed in ImageJ using the Cellcounter plugin (NIH).

QUANTIFICATION AND STATISTICAL ANALYSIS

Ca^{2+} imaging data analysis

Changes in Ca^{2+} fluorescence around active nose-pokes were analyzed as described previously (Jennings et al., 2015). Fluorescent signals from each cell were binned (window: −10 s before to 20 s after response) during Ext1 or Reinst sessions in MATLAB, smoothened (using a running 10-point moving average filter) and normalized (zscore) to the average activity for each trace within that window. Ca^{2+} activity was then averaged across all trials (nose-pokes) within a session for each cell. During extinction, only nose-pokes separated by at least 20 s from each other were used in analyses to prevent cross-contamination of signals between responses. During cued seeking, only nose-pokes occurring outside of the 20 s time-out between cue presentations (e.g., only cued nose-pokes) were evaluated. For the analysis of changes in population activity around nose-pokes, Ca^{2+} fluorescence signals were transformed to absolute values to incorporate both increased and decreased responses (Moorman and Aston-Jones, 2015). To quantify the population response magnitude, average Ca^{2+} activity was compared before (−2 to 0 s) and after (0 to 2 s) a nose-poke for each cell. To calculate the proportion of cells with increased or decreased responses to nose-pokes, non-absolute transformed Ca^{2+} signals within these same time-windows were compared and significantly modulated cells were defined as having average activity > 2 s.d. higher (increased) or > 2 s.d. lower than baseline prior to a nose-poke.

Histology clustering analysis

To investigate whether the different neuronal populations were organized as clusters or randomly distributed throughout the VP, we used a k-nearest neighbor algorithm (k = 10) on the centroids (ImageJ Cellcounter) of neurons identified by either virus expression (in Cre mouse lines) or *in situ* hybridization for the different VP cell markers. The k-nearest neighbor value (Euclidian distance) for all cells was compared to the k-nearest neighbor values of a shuffled dataset (containing the same number of randomly distributed datapoints, and repeated 1000 times).

Statistics

All data are presented as mean ± sem. Statistical analyses were performed using Prism (Graphpad; version 6.2). Paired Student's t tests were used for extinction tests. One sample t tests were used for clustering analyses. One-way and two-way repeated-measures analysis of variance (RM-ANOVA) with Greenhouse-Geisser correction and Neumann-Keuls post hoc tests were used for cue seeking tests and Ca^{2+} event rate and amplitude comparisons as specified in the results. Chi-square tests with Bonferroni correction for repeated-measures was used for rabies tracing data and Ca^{2+} population fraction comparisons. Statistical significance was set at 0.05.

DATA AND CODE AVAILABILITY

The published article includes all data generated or analyzed during this study. MATLAB code written by J.A.H. was used to process and analyze all calcium imaging datasets. Code, and data are openly available upon request.

Cell Reports, Volume *30*

Supplemental Information

Opposing Regulation of Cocaine Seeking

by Glutamate and GABA Neurons

in the Ventral Pallidum

Jasper A. Heinsbroek, Ana-Clara Bobadilla, Eric Dereschewitz, Ahlem Assali, Reda M. Chalhoub, Christopher W. Cowan, and Peter W. Kalivas

SUPPLEMENTAL INFORMATION

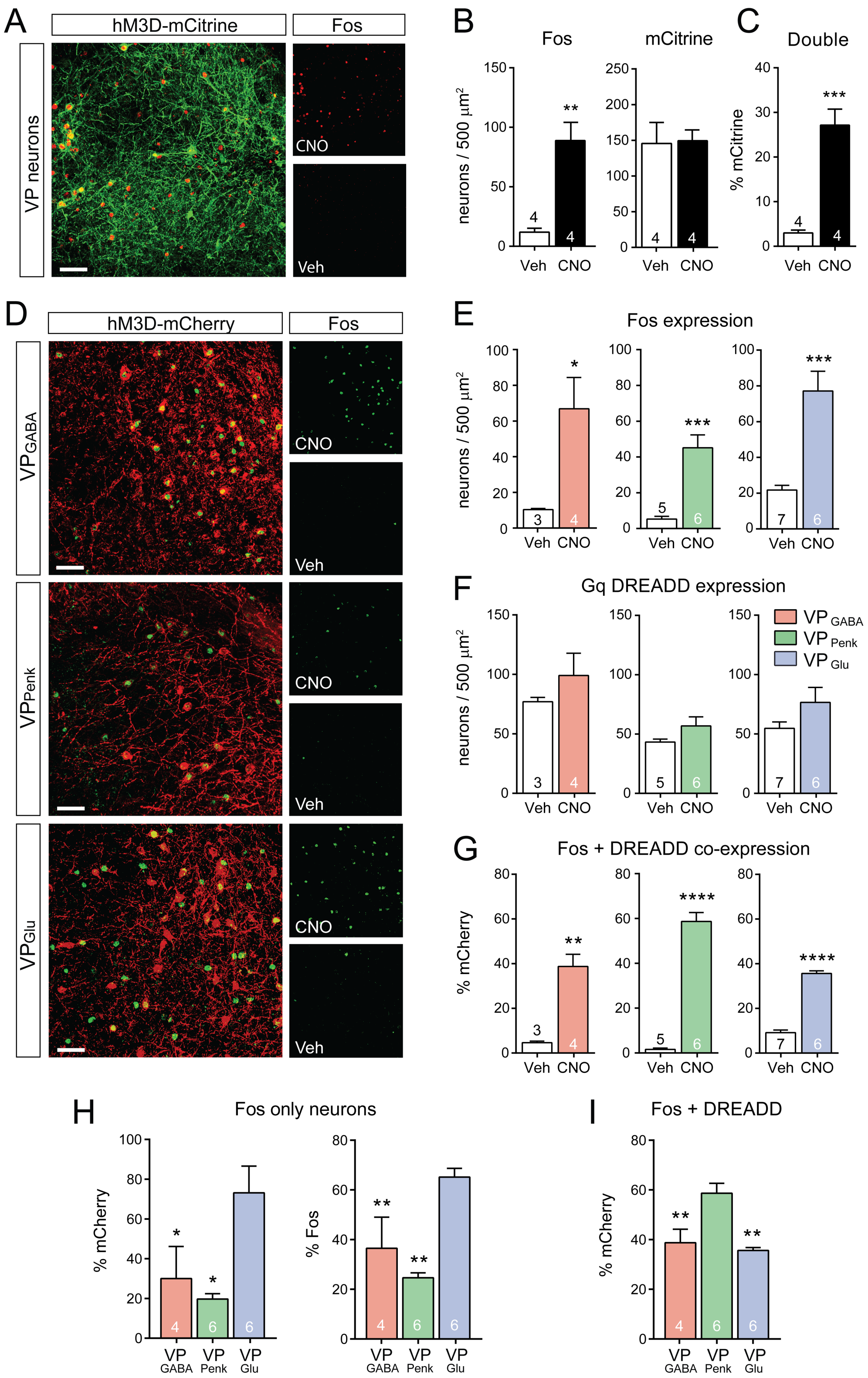

Figure S1. Gq DREADD Activation by CNO Induces Fos in VP Neurons, Related to Figure 2 and 3. (A) Representative micrograph showing that Fos expression induced by CNO co-localizes with Gq DREADD (mCitrine) expression in the VP. Inserts to the right show Fos expression following an injection of CNO (top) or vehicle (Veh, bottom). Bar= 50 um. (B) CNO significantly induces Fos in the VP of animals infected with Gq DREADD. (C) CNO induces Fos in Gq DREADD virus (mCitrine) expressing neurons. (D) Representative images showing Fos and Gq DREADD co-expression in VP_{GABA}, VP_{Penk} and VP_{Glu}. Inserts on the right show Fos induction in an animal injected with CNO (top) or Veh (bottom). Bar= 50 um. (E) Gq DREADD activation significantly induced Fos in all VP neuronal subtypes. (F) Gq DREADD (mCherry) expression levels were similar between CNO and Veh treated mice. (G) CNO significantly induced Fos in virus infected neurons across all cell-type. (H) Non-virus infected neurons were Fos activated in significantly higher numbers following stimulation of VP_{Glu} compared to VP_{GABA} or VP_{Penk} neurons (counts normalized to total virus expression or total Fos expression). (I) CNO induces Fos in a larger fraction of VP_{Penk} compared to VP_{GABA} or VP_{Glu} neurons. n in bars = number of animals. Data represented as mean ± SEM. * $p<0.05$, ** $p<0.01$, *** $p<0.001$, **** $p<0.0001$ (for H comparing VP_{GABA} and VP_{Penk} to VP_{Glu}, for I comparing VP_{GABA} and VP_{Glu} to VP_{Penk}).

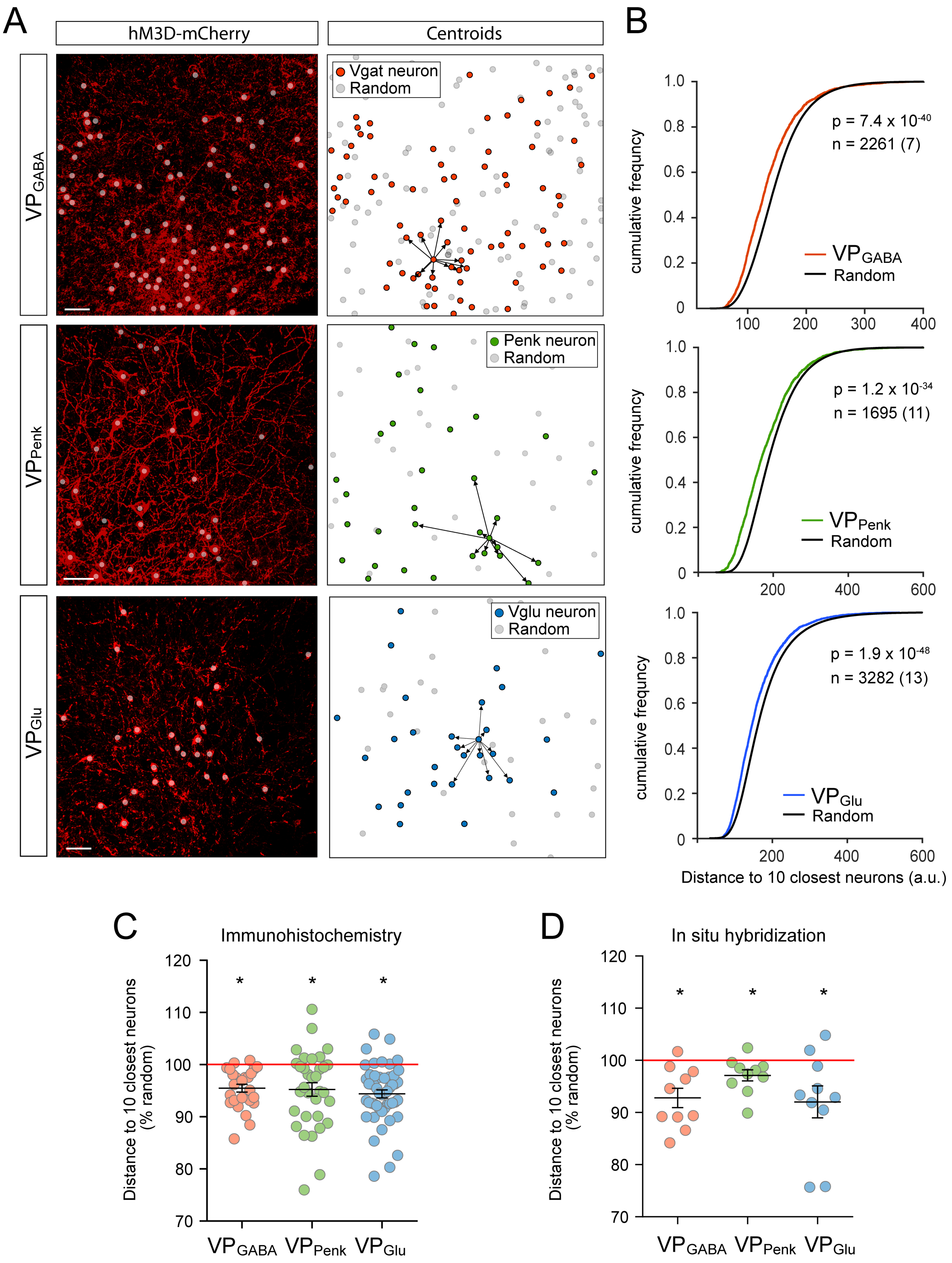
A
hM3D-mCherry
Centroids
VP_{GABA}
VP_{Penk}
VP_{Glu}
Vgat neuron
Random
Penk neuron
Random
Vglu neuron
Random
B
cumulative frequncy
$p = 7.4 \times 10^{-40}$
n = 2261 (7)
VP_{GABA}
Random
$p = 1.2 \times 10^{-34}$
n = 1695 (11)
VP_{Penk}
Random
$p = 1.9 \times 10^{-48}$
n = 3282 (13)
VP_{Glu}
Random
Distance to 10 closest neurons (a.u.)
C
Immunohistochemistry
Distance to 10 closest neurons (% random)
D
In situ hybridization

Figure S2. VP Subpopulations are Organized in Clusters, Related to Figure 3. (A) Left: micrographs showing mCherry expression in subpopulations of VP neurons with overlaid centroids for each cell (white dots). Right: Example centroid maps (color) for representative image stacks of each cell type combined with a randomly generated centroid map (grey) containing the same number of datapoints, as well as an example showing the 10 nearest neighbors of a single cell. (B) Cumulative frequency distribution for the distances of each VP cell to its 10 nearest neighbors, and the equivalent distance between random datapoints. VP subpopulations were significantly more clustered than random data (2-sample Kolgomorov-Smirnov test) n= total cell# over (mouse #) collected in 3-4 image stacks per animal. (C) K-nearest neighbor distance for all cells per image stack (normalized to the equivalent distance between random datapoints in that stack). VP neurons identified by mCherry expression (D) or RNA expression (E) were significantly more organized as clusters than random data (one-sample t-test). Error bars represent SEM. * $p < 0.05$

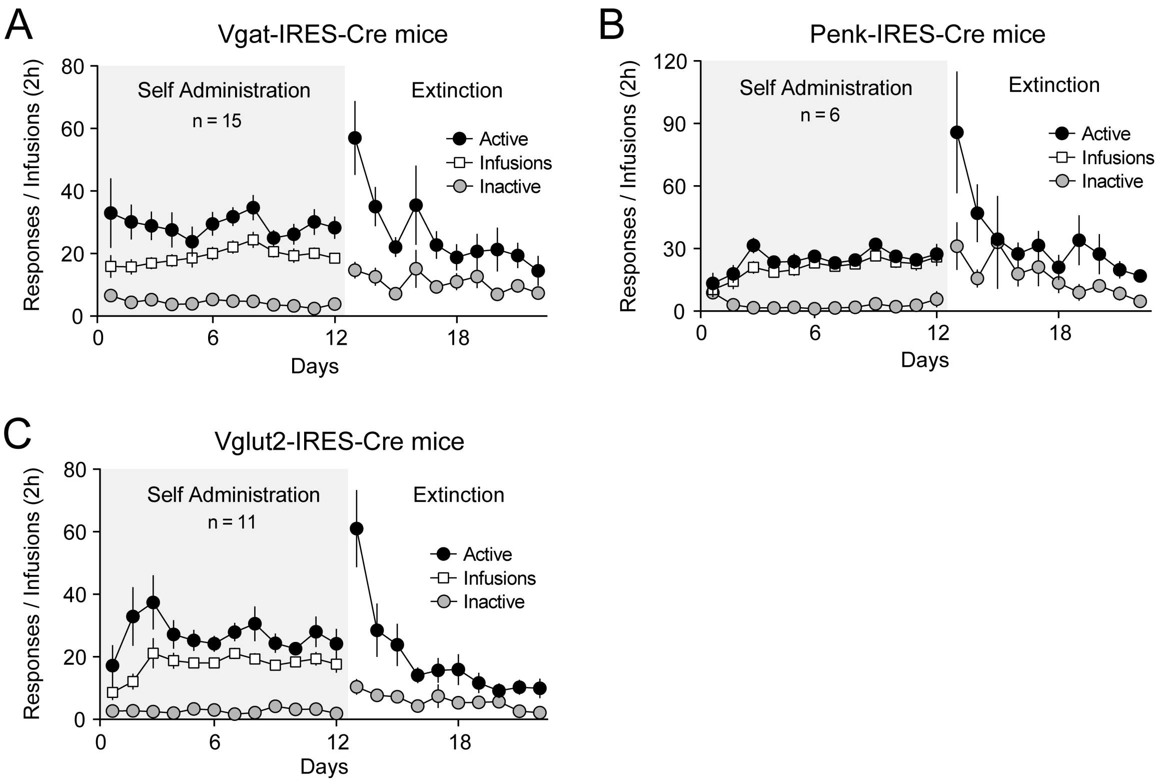

Figure S3. Cocaine Self-administration and Extinction for Gq DREADD Experiment, Related to Figure 3. (A) Vgat-IRES-Cre mice, (B) Penk-IRES-Cre mice, and (C) Vglut2-IRES-Cre lines acquired stable self-administration after 3d and extinguished operant responding in the absence of cocaine. Data represented as mean ± SEM.

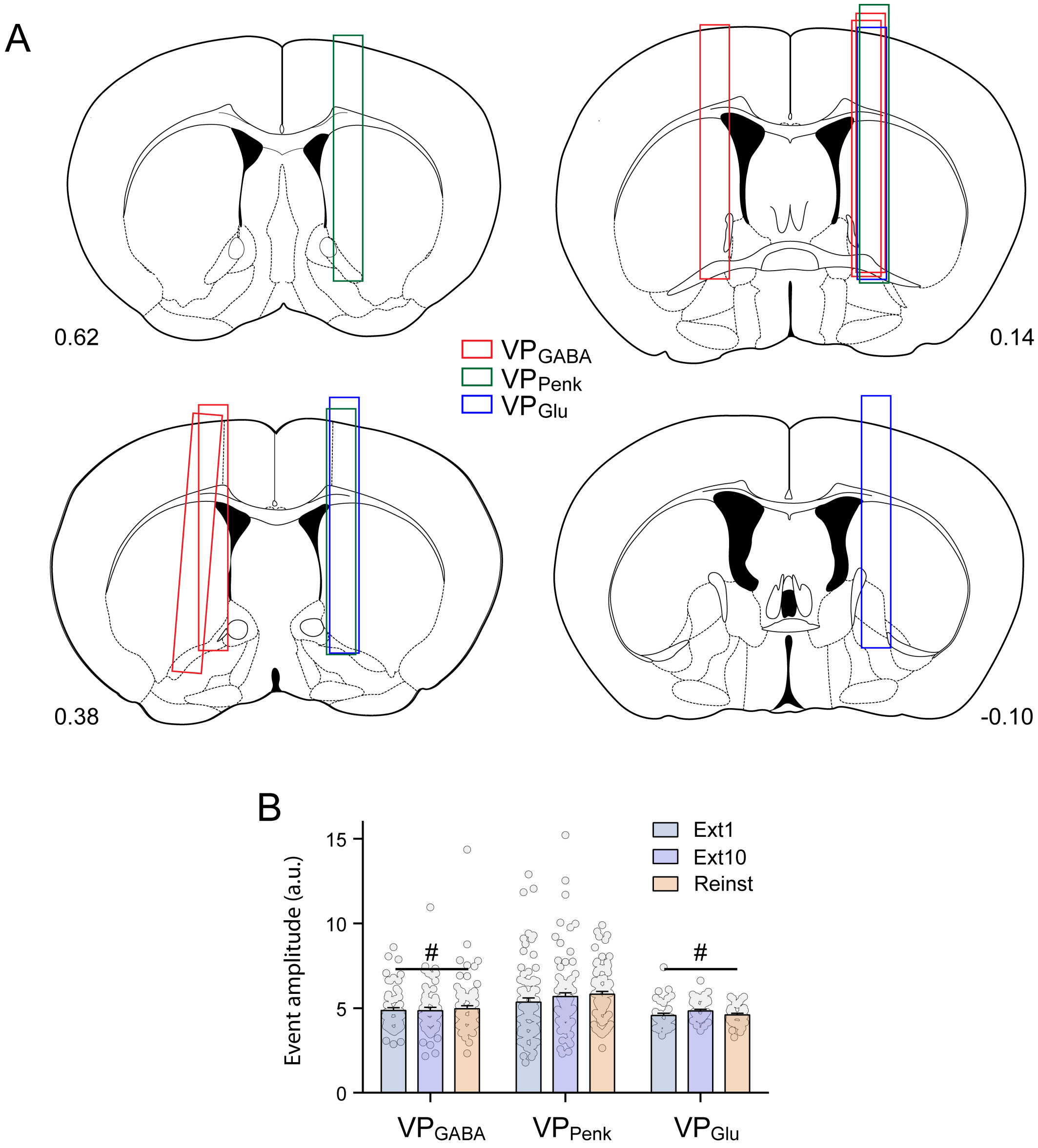

Figure S4. GRIN Lens Placements and Ca^{2+} Event Amplitudes, Related to Figure 4 and 5. (A) Placements of GRIN lenses for recordings of the different VP subpopulations of neurons. Numbers represent anteroposterior coordinates relative to Bregma. (C) Ca^{2+} event amplitude does not differ across conditions, but the Ca^{2+} event amplitude of VP_{Penk} neurons is significantly higher than that of VP_{Glu} and VP_{GABA} neurons. Data represented as mean ± SEM. # $p < 0.05$ compared to VP_{Penk}.

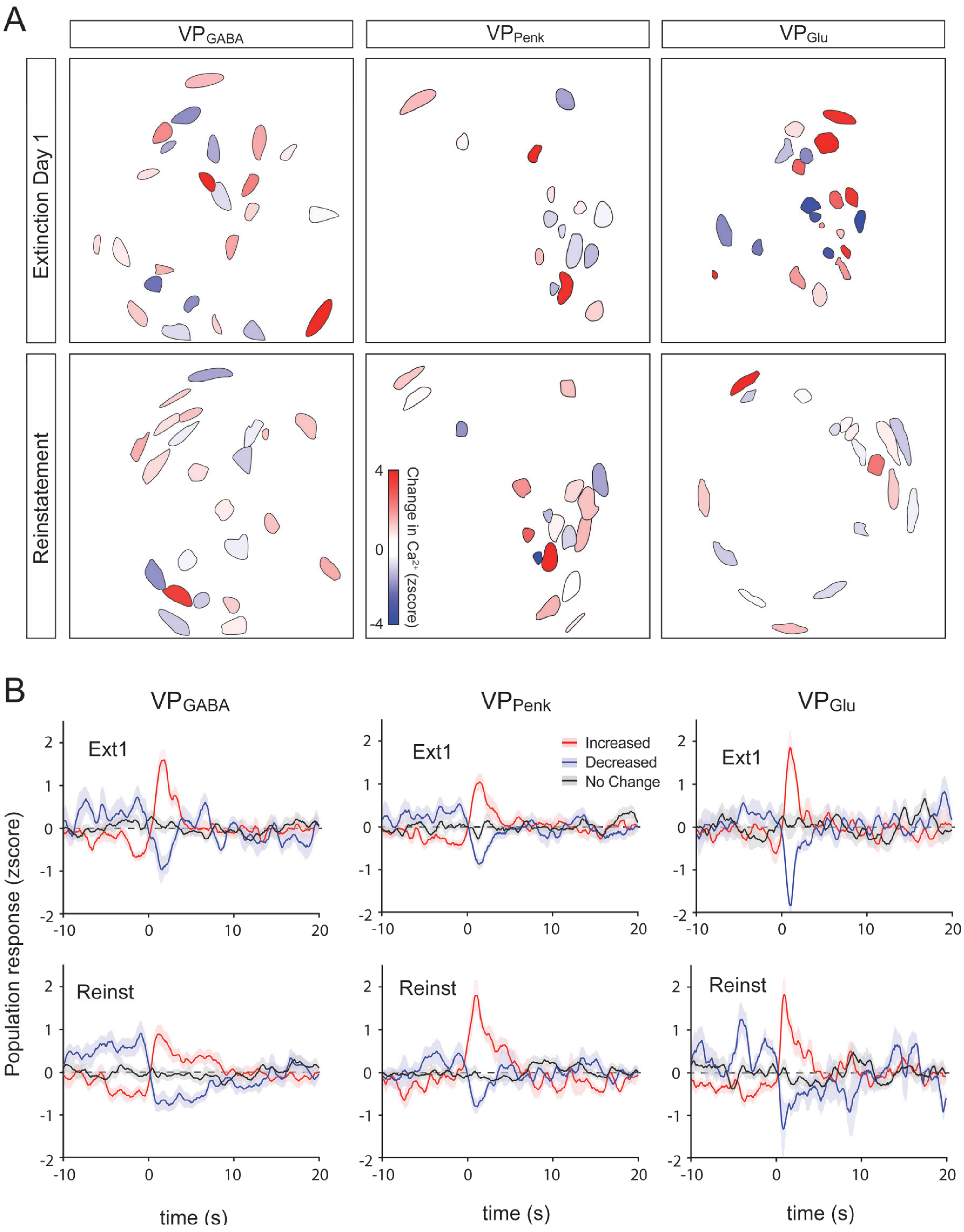
A
VPGABA
VPPenk
VPGlu
Extinction Day 1
Reinstatement
4
0
-4
Change in Ca2+ (zscore)
B
VPGABA
VPPenk
VPGlu
Ext1
Reinst
Increased
Decreased
No Change
Population response (zscore)
time (s)
2
1
0
-1
-2
-10
0
10
20

Figure S5. Populations of VP Neurons Subdivided by Increased or Decreased Activity Patterns, Related to Figure 5. (A) Example cell maps of the distinct VP neurons isolated from Ca^{2+} videos, pseudocolored by activity profile showing neurons responding with increased or decreased activity during the first 2s following a nosepoke are spread heterogeneously throughout the VP. (B) Population responses for all cell types and conditions separated by neurons that show increased, decreased or unchanged Ca^{2+} activity 2s following a nosepoke. Data represented as mean (lines) ± SEM (shading).
